# Mechanisms and therapeutics of immunometabolic reprogramming driving macrophage-ECs interactions in sepsis-associated ARDS from the gut-lung axis perspective

**DOI:** 10.3389/fimmu.2026.1730799

**Published:** 2026-03-02

**Authors:** Jia Tang, Mi Yan, Yanfei Liu, Wanwei Li, Zhangxue Hu

**Affiliations:** Department of Pediatrics, Daping Hospital, Army Medical University, Chongqing, China

**Keywords:** sepsis, ARDS, gut-lung axis, immunometabolic reprogramming, macrophages, ECS, cell interaction, inflammatory response

## Abstract

Acute respiratory distress (ARDS) caused by sepsis is a critical inflammatory condition with high mortality rates in clinical settings. The gut-lung axis plays a crucial role in regulating the immune response in both the intestinal and pulmonary environments, significantly impacting the development of ARDS. Immunometabolic reprogramming, a fundamental regulator of immune cell function, has recently been shown to profoundly affect the activity of macrophages and endothelial cells (ECs), as well as their crosstalk, thereby shaping the pathogenesis of ARDS. While a great deal has been learned about the potential inflammatory pathways involved, few clinically actionable therapies are available in part due to an incomplete understanding of gut-lung crosstalk in their shared ecosystem of cells and molecules. The current review systematically advances novel insights into the immunometabolic reprogramming that influences macrophage-ECs interactions via sepsis-induced ARDS, with a specific regard to the gut-lung axis. Here, we summarize the key biochemical pathways that control immune cell phenotypes and endothelial function, review the latest experimental evidence for their intercellular crosstalk, and describe the molecular targets that might be targeted to inform therapeutic strategies. Integrating the current evidence, this review seeks to provide a comprehensive theoretical framework and novel methods for the precise treatment of sepsis-associated ARDS, which could be beneficial to clinical practices and patients’ prognoses.

## 1 Introduction

Sepsis-induced ARDS is one of the most challenging issues in critical care medicine due to its high morbidity rate, complex pathogenesis, and limited effective treatments. ARDS is characterized by sudden extravascular lung inflammation that leads to loss of alveolar-capillary membrane integrity, pulmonary edema, and severe hypoxemia. When combined with sepsis, a form of sepsis-induced lung injury, the prognosis worsens significantly, and the high mortality rate persists despite advances in supportive care, including protective mechanical ventilation and fluid management (1). The ability to predict and intervene in these patients remains poor, making clinical treatment particularly challenging. Biomarkers such as presepsin have been identified as promising tools for risk stratification and diagnosis of sepsis-mediated ARDS (2).

The development of sepsis-related ARDS implicates intertwined systemic inflammation, immune dysregulation, and cell injury within the lung microenvironment. Interestingly, the gut-lung axis has been identified as a clinically important immunoregulatory pathway connecting the intestinal microbiota and pulmonary immune system to impact on ARDS pathogenesis. The microbial community of the gut influences systemic immunity and inflammation through microbial metabolite products and through interactions with immune cells; dysbiosis of the gut flora during sepsis can drive lung injury. The concept of fecal microbiota transplantation (FMT) in the ARDS model group suggests that rebalancing gut microbiota may mitigate lung injury, improve pulmonary function, and regulate immune/metabolic pathways, highlighting the value of targeting the gut-lung axis (3). Furthermore, alterations in the composition and functionality of gut microbiota have been associated with the severity of COVID-19-induced ARDS, thus highlighting the more general involvement of gut–lung crosstalk in inflammatory lung syndromes (4). An important second hit that contributes to immune dysfunction in sepsis-induced ARDS is immunometabolic reprogramming, which regulates the functional states of both immune and ECs within the lung microvasculature. Immune cells, especially macrophages, undergo metabolic reprogramming that affects their polarization, the production of inflammatory mediators, and the phagocytosis of ECs.

Pathway targeting of metabolic processes that include mitochondrial antiviral-signaling proteins and interferon-regulatory factors drives M1 macrophage polarization with a proinflammatory phenotype, leading to augmented lung injury in sepsis-induced ARDS (5). At the same time, ECs undergo reprogramming of their metabolic and functional states, characterized by increased permeability and the expression of adhesion molecules, which contribute to the vascular leakage/leukocyte infiltration observed in ARDS (6). These metabolic changes are closely linked to inflammatory signaling pathways, Reactive oxygen species (ROS) generation, and cellular energy requirements, which collectively determine the leukocyte landscape in the injured lung (7). Macrophage-ECs communication contributes to the augmentation of inflammation and permeability in ARDS. Activated macrophages secrete cytokines and chemokines that cause endothelial activation, whereas the endothelium in turn regulates the recruitment and function of macrophages. In the era of advanced *in vitro* models, from lung endothelial microphysiological systems to more recent studies, it has been demonstrated how plasma from patients with sepsis can cause endothelium-dependent dysfunctions showing metabolic- and inflammation-mediated crosstalk (6).

Furthermore, mucin1(MUC1) and Ankyrin Repeat Domain 22 (ANKRD22) have been reported as molecular mediators in the regulation of macrophage polarization and administration of endothelial response, suggesting potential therapeutic targets for attenuation of sepsis-induced lung injury (8).In consideration of the critical role of immunometabolic reprogramming in driving macrophage-endothelial interplay and lung injury, an extensive discussion related to the above mechanism from microbiota-lung axis viewpoints is crucial. This extensive review highlights how the dysbiotic gut microbiota influences systemic immunometabolism of conventional organs and pulmonary tissues, while steering the cellular crosstalk necessary for promoting ARDS responsiveness. More recently, multi-omics studies have described unique metabolic and immune phenotypes that distinguish different putative sub-phenotypes of sepsis-induced ARDS, leaning towards precision medicine (9, 10).

**Figure 1:**
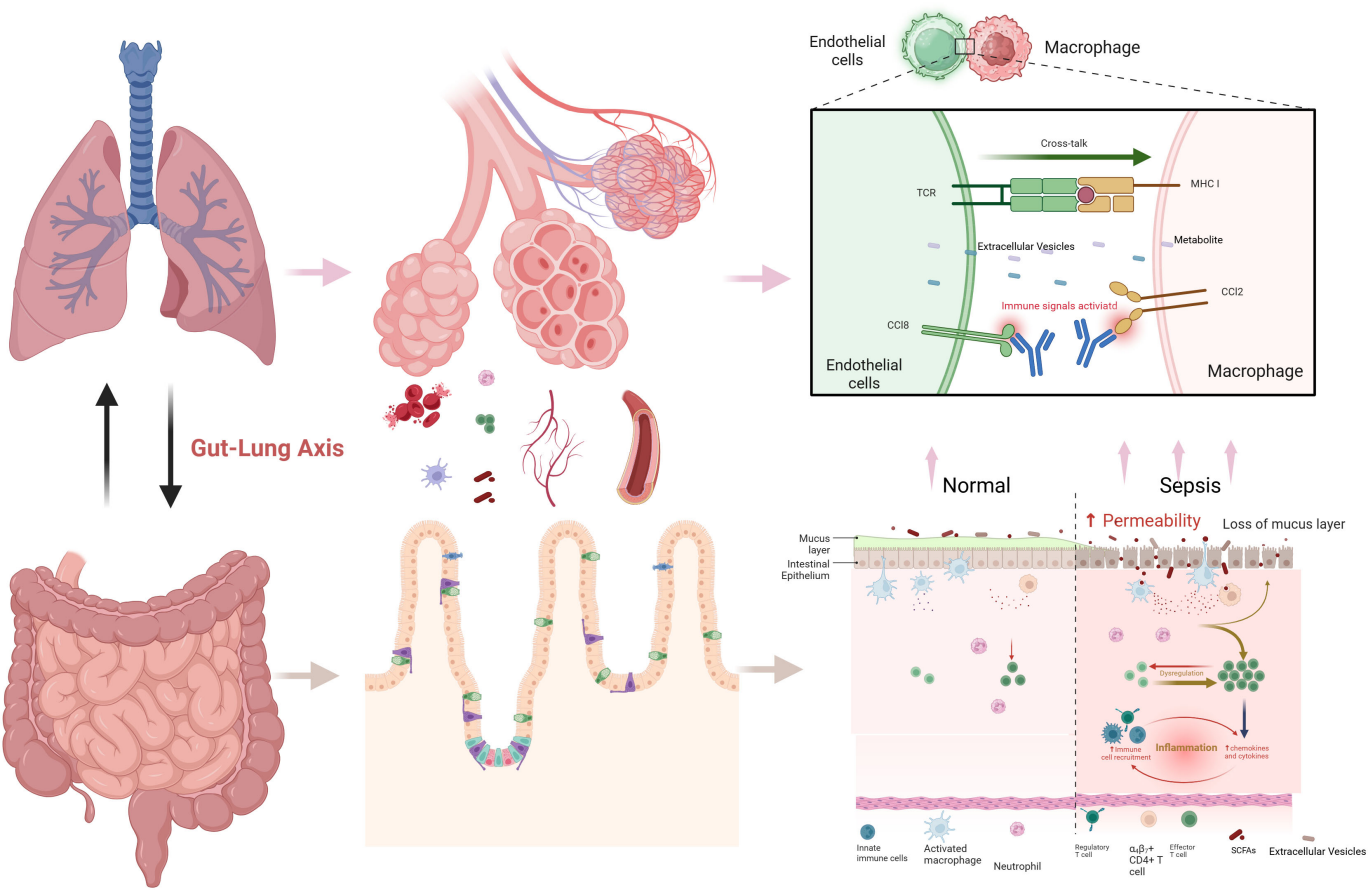
Macrophage-ECs interactions in sepsis-associated ARDS through gut-lung axis. [Created with BioRender.com (11)].

The metabolic pathways regulating macrophage and ECs function in the context of the gut-lung axis are promising targets for therapeutic approaches to improve sepsis-associated ARDS outcomes. This review seeks to systematically summarize our contemporary understanding of how immunometabolic reprogramming-mediated macrophage-ECs interplay may function in sepsis-related ARDS and highlights the regulatory role of the gut-lung axis. By combining findings from biomarker studies, cellular and molecular approaches, and pre-clinical models, we aim to identify novel therapeutic targets and approaches that may alter the management of this syndrome (**Figure 1**).

## 2 Gut-lung axis

### 2.1 Pathophysiological basis of sepsis-induced ARDS and the role of the mechanism of the gut-lung axis

#### 2.1.1 Clinical features and pathological mechanisms of sepsis-associated ARDS

Sepsis-induced ARDS is a life-threatening disease that is caused by an acute inflammatory response and results in serious pulmonary function damage and high lethality. Clinically, sepsis-induced ARDS is characterized by the acute inflammatory disruption of the alveolocapillary barrier, which leads to elevated permeability with subsequent pulmonary edema and impaired gas exchange. The pathophysiology of alveolar-capillary barrier dysfunction is mainly derived from an exaggerated inflammatory response leading to endothelial and epithelial lung injury. This injury results in the accumulation of proteinaceous fluid in the alveoli, leading to hypoxia and pulmonary insufficiency. It is well-documented that the outcome of patients with sepsis-induced pulmonary ARDS seems to be worse than that of extrapulmonary ARDS; this also confirms the more severe lung involvement in patients with sepsis (12). Inflammatory cytokines such as interleukin-6 (IL-6), interleukin-8(IL-8), and tumor necrosis factor-α (TNF-α) are released in the early phase of inflammation, leading to enhanced activation and permeability of the endothelium, which contributes to lung injury (13). Systemic inflammation during sepsis results in the release of a variety of proinflammatory mediators in circulation that not only affect the lungs but also cause multiorgan dysfunction. The lung is one of the most frequently affected organs because of its rich microvascular system and direct exposure to inflammatory cells and mediators in circulation. Endothelial impairment is a typical feature of sepsis-induced ARDS, including upregulation of adhesion molecules such as intercellular adhesion molecule-1(ICAM-1), breakdown of endothelial junctions, and stimulation of leukocyte infiltration into the lungs. This endothelial damage leads to pulmonary edema and hypoxemia, which are classic features of ARDS. Another one is the imbalance of the Angiopoietin-Tie2 signaling pathway, which plays a key role in maintaining the integrity of microvessels, sepsis-induced angiopoietin imbalances associated with increased permeability and microthrombosis in pulmonary circulatory (14). Associated pulmonary oedema and alveolar flooding disrupt lung mechanics and result in hypoxemia and respiratory failure. Molecular profiling has shown significant metabolic and transcriptomic differences in sepsis-induced ARDS. Metabonomics showed that the metabolic fingerprints of sepsis-induced ARDS were significantly different from those of control and non-ARDS, which might be related to the disturbance in glycerophospholipid and sphingolipid metabolism pathways. Furthermore, in ARDS, transcriptome profiling showed deregulated genes involved in immune response and metabolic processes, suggesting a complex interaction between dysregulated immune response and metabolism leading to its pathogenesis (15, 16). Immune cell profiling reveals changes in monocytes, neutrophils, and macrophages circulation, with macrophage M1 polarization being a key determinant for sustaining lung inflammation and injury. Furthermore, disordered autophagy and ER stress pathways have been involved in the pathogenesis of sepsis-induced ARDS with potential therapeutic targets (17, 18).

Clinically, sepsis-induced ARDS is characterized by hypoxemia, decreased pulmonary compliance, and bilateral infiltrates on the chest radiographs consistent with non-cardiogenic pulmonary edema. Worse outcomes and higher mortality are associated with the severity of hypoxemia, commonly assessed by the PaO_2_/FiO_2_ ratio 20. Clinical factors such as body mass index, respiratory rate, blood urea nitrogen, and immune cell count were used for model construction by machine learning algorithms to distinguish sepsis patients who are at high risk of ARDS so that treatment can be conducted early (19, 20). The potential of markers such as prespsin, MUC1, NOD-like receptor family (NLR) pyrin domain-containing 3 (NLRP3), and multidrug resistance proteins(Mrp) estimate the occurrence and outcome ARDS in sepsis (21, 22). Insight into the relationships between immune activation, metabolism rerouting, and endothelial injury is essential further to improve the diagnosis and treatment of this disastrous syndrome.

#### 2.1.2 Anatomical and functional connections of the gut-lung axis

The gut-lung axis is a complex network comprising the interconnections between the gastrointestinal tract (GIT) and lungs, facilitated by the immune system, metabolites, and anatomical structures. Central to this axis is the GI barrier, loss of integrity of which can lead to bacterial and their metabolites from the intestine into systemic circulation, possibly affecting the pulmonary immune context. When there is a breach of the gut as a barrier, called “leaky gut”, microbial components such as bacterial lipopolysaccharide (LPS) and other pathogen-associated molecular patterns (PAMPs) can spill into the circulation and travel via the portal vein and mesenteric lymphatic straight to the lung. It has been demonstrated that this translocation of microbes leads to lung inflammation, alters the homing of immune cells, and exacerbates respiratory diseases in ARDS (23). Common mucosal immune structures may reinforce the anatomic connections that program host responses in gut and lung mucosa, with the potential for the migration of immune cells and cytokines, which could mediate homeostatic balance or pathogen defense (24, 25). Functionally, the gut microbiota and its metabolites play a pivotal role in modulating lung immunity. Gut microbial communities produce bioactive metabolites—including short-chain fatty acids (SCFAs), tryptophan derivatives, polyamines, and secondary bile acids—that enter the circulation and exert systemic immunomodulatory effects. SCFAs, in particular butyrate and propionate, have been found to impact the metabolic programming and cytokine production of alveolar macrophages, thus defining the immune tone as well as inflammatory output of the lung (26–28). These metabolites exert their effects via receptors, such as free fatty acid receptors (FFAR)2 and FFAR3, or on various pathways, including histone deacetylase inhibition and G-protein coupled receptor signaling, which mediate the differentiation of regulatory T cells and suppress exaggerated inflammation within the lung (29). Furthermore, gut dysbiosis has been associated with impaired lung immunity and enhanced susceptibility to respiratory infections, involving low levels of microbial diversity and changes in composition (30–32).

Most microbial metabolites and immune mediators are distributed via the bloodstream and lymphatic system, carrying gut-derived signals to the lung. In addition, immune cell migration in and out of the gut mucosa occurs, with both lymphocytes and innate lymphoid cells (ILCs) contributing to immunosurveillance and regulation of inflammation within the lung. ILC2s show tissue-restricted maturation and migration along the lung-gut axis, controlling the mucosal immunity of these two organs (33). The portal vein and mesenteric lymphatics serve as key anatomical channels for this cross-talk, as they facilitate the transport of microbial products and immune cells, which can potentially modulate lung immune homeostasis (34). In sepsis-induced ARDS, acute lung injury (ALI), and other pathological conditions, gut barrier breach and microbial metabolic dysregulation result in systemic inflammation and deregulated immunity in the lungs. This is illustrated by enhanced bacterial translocation, augmented proinflammatory cytokine secretion, and immune cell mis-homing from the gut to the lung (23, 25). Environmental determinants, including PM2.5 and pollutants, can also disrupt gut and lung microbiota, thereby potentiating inflammatory cascades along the gut–lung axis. On the other hand, treatments related to gut microbiota, including probiotics, prebiotics, dietary fiber supplementation, and traditional Chinese medicine, have been reported for their therapeutic effects in recovering eubiosis of gut microbiota and improving the integrity of the barrier as well as lung inflammation with a modulation on the gut-lung axis (35, 36).

Anatomic and functional connections of the gut-lung axis are provided via intact intestinal barrier function, including systemic translocation of microbial metabolites and immunoactive factors, as well as trafficking of immune cells throughout mucosal sites. This axis plays a critical role in mediating pulmonary immune homeostasis and provides an essential mechanistic conduit by which perturbations of the composition or integrity of intestinal microbiota influence health and disease within the lung. These additional insights help to provide potential avenues for new therapeutic interventions targeting the gut microbiota and its metabolites in the prevention or treatment of respiratory diseases associated with immune and metabolic dysregulation.

#### 2.1.3 Pathological role of the gut-lung axis in sepsis-associated ARDS

The gut-lung axis is one of the major pathogenic mechanisms in sepsis-related ARDS, inducing GI barrier loss and lung inflammation. It is the systemic inflammatory response and the toxicity of microbial products, as well as enzyme-mediated attack, that contribute to increased gut permeability in sepsis. This disruption can be conducive to the translocation of bacterial endotoxins and proinflammatory mediators via gut into systemic circulation, lymphatic, and eventually go to the lung along with a gut-lymph-lung axis. The arrival of these gut-derived factors exacerbates lung injury by increasing alveolar-capillary barrier permeability, facilitating neutrophilic invasion, and triggering a cytokine storm, thereby hastening the onset of ARDS. Acute pancreatitis is a common etiology causing systemic inflammation and also sepsis to occur; the gut barrier dysfunction is important, where pancreatic enzymes and bacterial products further complicate lung injury, resulting in ARDS. Despite some progress in our understanding of this axis, studies have shown that no successful therapies are known to target the maintenance of gut barrier integrity as a means to combat ARDS, emphasizing the importance of integrated therapeutic approaches between gastroenterologists and intensivists to reduce organ failure and improve patient prognosis (37).

In addition to barrier breakdown, proinflammatory gut products enter the circulation and act directly on immune cells as well as ECs in the lung that play a crucial role in ARDS pathophysiology. Sepsis induces the recruitment of specific immune cells from the gut, such as γδ T17 in this case, to the lung and triggers localized IL-17A-dominant inflammation. This phenomenon is driven by the induction of Wnt signaling in alveolar macrophages, which subsequently induces the expression of chemokines, like C-C Motif Chemokine Ligand 1(CCL1), that dictate the migration of gut-immune cells to pulmonary tissues. The engrafted γδ T17 cells contribute to lung injury by maintaining local cytokine secretion and recruiting other immune cells, providing a direct cellular bridge between the gut immune disorder and pulmonary inflammation in sepsis-induced ARDS. Crucially, treatments that interfere with this signal pathway, e.g., esketamine, have been demonstrated to inhibit ARDS by preventing the gut-to-lung migration of immune cells, suggesting a way to deliver therapeutic approaches via the gut-lung immunity connection (38).

The gut-derived metabolic factors also affect lung immune and ECs function, which are involved in immunometabolic reprogramming during sepsis-related ARDS. Glucagon-like peptide-1 receptor agonists (GLP-1RAs), widely recognized for their role in glycemic control, have been identified as modulators of the gut-lung axis with potent anti-inflammatory and organ-protective properties. GLP-1RAs enhance gut barrier integrity and modulate the gut microbiota, thereby indirectly reducing systemic inflammation and preventing harmful metabolites from translocating into the circulation, where they can activate pulmonary macrophages and ECs. In the lung, they modulate ARDS by reducing the production of proinflammatory cytokines and improving the function of the alveolar-capillary barrier and surfactant secretion, in an effort to preserve lung structure and function. The pleiotropic effects of GLP-1RAs suggest a close relationship between metabolic signals from the gut mucosa and pulmonary immune responses, indicating that modulation of metabolic pathways within the gut-lung axis could be a potential therapeutic approach for attenuating sepsis-induced ARDS and improving outcomes in critical illness (39). In conclusion, the pathogenic functions of the gut-lung axis in sepsis-induced ARDS are multivariable, which include intestinal barrier damage and subsequent pulmonary inflammation amplification, mediated by direct migration of immune cells from the organ to the lung that contributes to lung injury, as well as gut-derived metabolic mediators that control immune cell function and ECs function within the lungs. A better understanding of these intricate mechanisms would lay the basis for new approaches to prevent ARDS by preserving the gut barrier, redirecting immune cell trafficking, and manipulating metabolic controls to mitigate ARDS severity and improve survival in patients with severe sepsis.

### 2.2 Basic concepts of immunometabolic reprogramming and its role in immune cells

#### 2.2.1 Definition and classification of immunometabolic reprogramming

Metabolic reprogramming and immunometabolism have been described as the changes that occur in energy metabolic pathways within immune cell types to govern cellular activation, polarization, and functional states. Immune cells exhibit exceptional metabolic plasticity and adaptability, allowing them to adjust their metabolic fluxes in response to both environmental cues and immunological challenges. Oxidative phosphorylation (OXPHOS) is the primary energy producer in immune cells at rest; however, upon stimulation, many immune cell populations shift from a quiescent OXPHOS preference to an activated glycolytic phenotype, characterized by a heightened ability to generate adenosine triphosphate (ATP) and biosynthetic products required for effector function. None of this metabolic crosstalk results from immune activation, but controls the destiny and function of these immune cells. ATP is required for proliferation, cytokine secretion, and the glycolysis products favor nucleotide and lipid synthesis, whereas OXPHOS often supports anti-inflammatory or regulatory phenotypes, including M2 macrophages or regulatory T cells. Fatty acid metabolism also significantly influences immune responses in multiple immunological phenomena by controlling cell survival, differentiation, and effector functions. The classification of immunometabolic reprogramming can be delineated based on predominant metabolic pathways engaged during immune responses. Proinflammatory immune cells, including M1 macrophages and effector T cells, predominantly rely on aerobic glycolysis—a phenomenon reminiscent of the Warburg effect observed in cancer cells—to meet their energetic and biosynthetic needs. Conversely, anti-inflammatory or regulatory immune cells, including M2 macrophages and memory T cells, preferentially utilize OXPHOS and fatty acid oxidation (FAO), which support long-term survival and function. This metabolic dichotomy underpins the functional polarization of immune cells and is tightly regulated by signaling pathways, including phosphatidylinositol 3-kinase (PI3K)/active protein kinase C-α (Akt)/mammalian target of rapamycin (mTOR), Hypoxia-inducible factor (HIF-1α), and adenosine 5’-monophosphate (AMP)-activated protein kinase (AMPK).

Recent research also extends the concept of immunometabolic reprogramming, not limited to glucose, but including amino acids and lipids that fine-tune immune cell phenotypes. Arginine metabolism is implicated in trained immunity and macrophage activation (34), while lipid metabolites can function as signaling molecules that modulate immune cell activity and inflammatory processes. The metabolism of immune cells is also regulated by the microenvironment, including nutrient and oxygen availability, and that may induce an adaptive response that impacts disease progression and therapy response. Immunometabolic rewiring is involved in the pathogenesis of a myriad of diseases, including infection, autoimmunity, cancer, and metabolic disorders. Metabolic imbalance of immune cells contributes to ineffective or excessive stimulation, inhibition of the activation, or an overt cellular suppression and eventually inflammation or escape from the immune surveillance. The observation that immune cells also exhibit particular metabolic patterns and regulations in various pathological contexts laid the foundation for immunotherapy targeting metabolic pathways to influence immunity. Approaches such as the inhibition of glycolysis to disable proinflammatory macrophages or the enhancement of FAO to fuel regulatory T cell function demonstrate the translational potential that targeting immunometabolism can bring.

Immunometabolic reprogramming refers to the dynamic and context-specific regulation of immune cell metabolism, mainly concerning glycolysis, OXPHOS, and FAO, among others, which control immune cell activation and polarization, playing immunoregulatory roles. The immunometabolic classification of immune cells is crucial in understanding immunometabolism in health and disease, and represents potential targets for immunometabolic therapy (40–44).

#### 2.2.2 Characteristics of macrophage metabolic reprogramming

Metabolic rewiring of macrophages is a critical determinant in their functional polarization towards phenotypically different states, especially M1 proinflammatory and M2 anti-inflammatory polarized states, displaying the metabolic preferences to support their specialized immune function and tissue homeostasis. (M1 ‘‘classically activated’’) Macrophages are induced by interferon gamma (IFN-γ) and LPS, which rely mostly on aerobic glycolysis, a metabolic pathway that allows for efficient ATP generation and provides biosynthetic intermediates necessary for the synthesis of inflammatory mediators (19, 20). This glycolytic shift is associated with perturbations in the tricarboxylic acid (TCA) cycle and mitochondrial OXPHOS fluxes, also resulting in the accumulation of downstream metabolites, including succinate and itaconate, which are additional modulators of inflammatory signaling that stabilize HIF-1α or inhibit succinate dehydrogenase, respectively. The metabolic reprogramming of M1 macrophages supports their abundant release of proinflammatory cytokines, as well as ROS production and NO generation, which are necessary to eliminate pathogens effectively but can have detrimental effects on tissues if unchecked (45–48).

**Figure 2:**
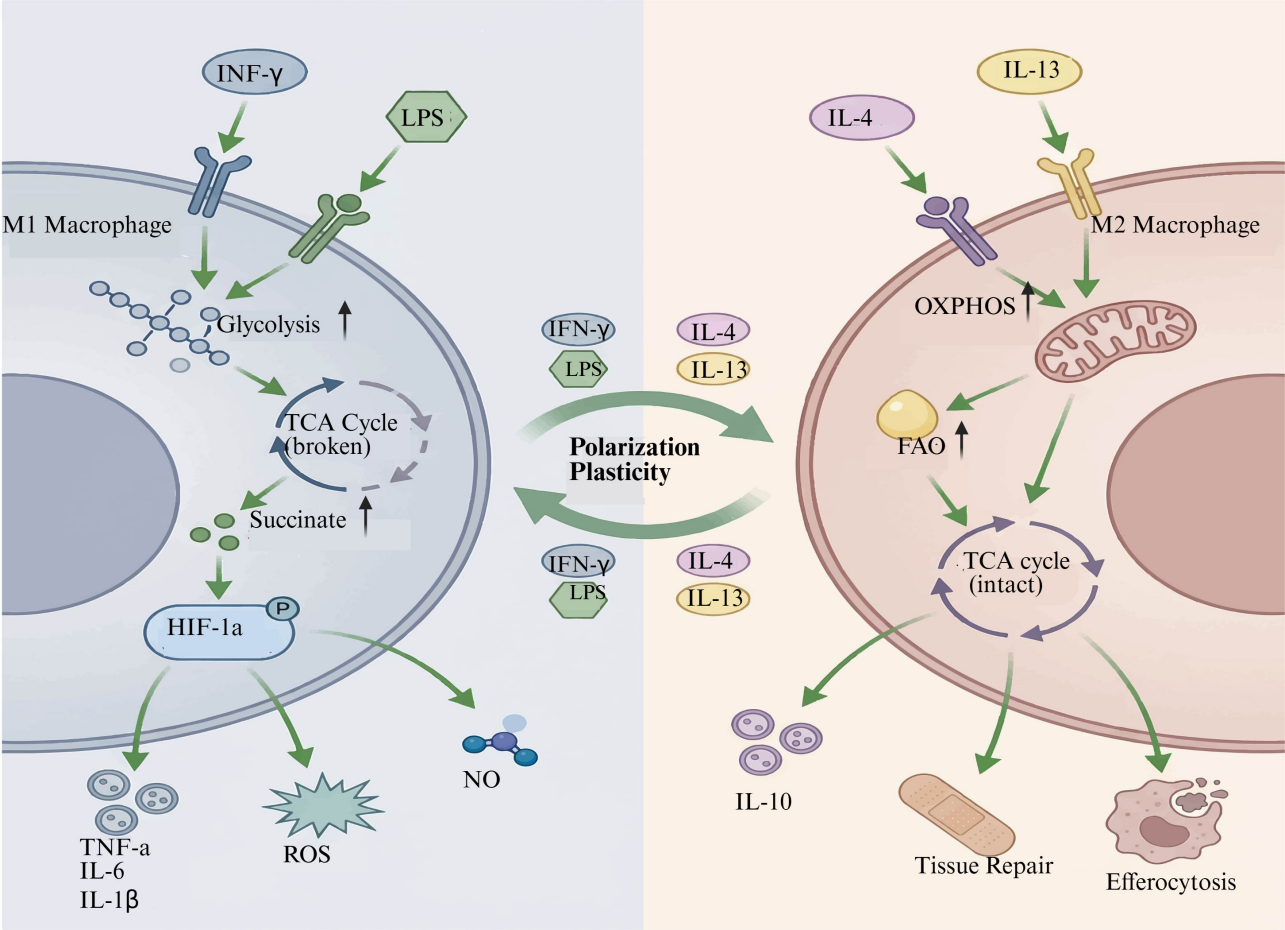
Immunometabolic reprogramming of macrophages in sepsis-induced ARDS. [Created with BioRender.com (11)].

In contrast, M2 macrophages rely predominantly on mitochondrial OXPHOS and FAO for their energy supply. This metabolic state helps them fulfill the roles of tissue repair, inflammation resolution, and immune regulation. M2 macrophages function as a normal TCA cycle and slightly enhanced mitochondrial respiration for anti-inflammatory cytokine production and extracellular matrix remodeling (**Figure 2**). It was recently demonstrated that the metabolic sensor serine/threonine kinase 11 (STK11) synchronizes IL-4 signaling with metabolic rewiring in M2 macrophages, limiting over-polarization by regulating glutamine metabolism and downstream transcription factors, including forkhead box O1 (FOXO1). Pharmacologic inhibition of the glutamine biosynthetic pathway or FOXO1 leads to the reversal of M2 polarization, emphasizing a strong connection between cellular metabolism and macrophage function (49). In addition to NLRP3 regulation, mitochondrial homeostasis and lipid metabolism are important for M2 macrophage function, as evidenced by peroxiredoxin 3 (PRDX3), which regulated the maintenance of mitochondrial integrity and exerted an anti-inflammatory effect; it also promoted M1 to M2 transition via a decrease in glycolysis and an enhancement of TCA cycle activity (50). The dichotomy in metabolic programming of M1 and M2 macrophages is not distinct; macrophages have a strong plasticity and potential to change their metabolism according to microenvironmental signals. Tumor-associated macrophages (TAMs), frequently showing an M2-like phenotype, reprogram metabolism in a complex manner, including the glucose, lipid, and amino acid metabolic circuits of cells to maintain their immunosuppressive functions within the tumor microenvironment (TME). TAMs enhance glycolysis and FAO, control cholesterol efflux, and modify amino acid pyrimidine biosynthesis, including arginine and tryptophan metabolism, to participate in tumor growth promotion and immune evasion (51). Likewise, in chronic infections or inflammatory disorders, such as rheumatoid arthritis and sepsis, macrophages exhibit metabolic rearrangements that shape their polarization and effector functions, demonstrating the relevance of metabolic plasticity for immune reactions (52–55).

The metabolic plasticity of macrophages is controlled by inner workings involving various biological signals and epigenetic modification. Master transcriptional regulators like HIF-1α, nuclear factor kappa B (NF-κB), and peroxisome proliferator-activated receptor γ coactivator 1 alpha (PGC1α) can sense metabolic signals to influence the expression pattern of macrophage polarization. HIF-1α stabilization in M1 macrophages leads to increased expression of glycolytic genes and production of proinflammatory cytokines, whereas PGC1α promotes mitochondrial biogenesis and OXPHOS in M2 macrophages (54–56). Moreover, metabolites generated during metabolic reprogramming can be signaling molecules that drive post-translational modifications and immunoreactivity. Itaconate, produced by the enzyme aconitate decarboxylase 1(ACOD1), is another example of such a metabolite that exerts anti-inflammatory activity via the inhibition of succinate dehydrogenase and activation of antioxidant pathways (48). Indeed, modulating macrophage bioenergetics is a critical process in the pathogenesis and resolution of diseases. LPS-activated M1-macrophages display enhanced glycolysis and impaired mitochondrial metabolism in sepsis, leading to systemic inflammation and tissue injury. Some pharmacological strategies, including metabolic reprogramming, glycolysis inhibition, or boosting mitochondria’s capacity, have proven effective in determining macrophage polarization and have worked well in experimental assays (57, 58).

Furthermore, recent studies show that extracellular components such as acid microenvironment and metabolites modulate macrophage metabolism and phenotype, which present other potential therapeutic targets (59). Overall, the specific metabolic reprogramming of macrophages is closely associated with their polarization states and roles. M1 macrophages rely on glycolysis, which is the primary energy source required for their anti-inflammatory functions, whereas M2 macrophages use OXPHOS and FAO to fuel tissue repair and immunoregulation. However, the molecular mechanisms that orchestrate these metabolic changes offer important insights into macrophage biology and may provide new targets for treating inflammatory diseases, infection, and cancer.

#### 2.2.3 ECs metabolic regulation and its functional impact

ECs have distinct metabolic features that are essential for regulating vascular homeostasis, and their own metabolism significantly impacts vessel permeability, inflammation, and cell viability. Under physiological conditions, ECs primarily rely on glycolysis in the presence of oxygen, which provides a necessary rapid adaptive ability for these cells to respond to environmental changes and meet their angiogenic requirements (60, 61). This glycolytic preference allows ECs to retain mitochondrial functionality for signaling rather than energy supply, which is essential both for its barrier function and vascular reactivity (62). However, alterations in endothelial metabolism, i.e., changes in glycolysis, FAO, and mitochondrial respiration, may lead to the occurrence of endothelial dysfunction associated with increased vascular permeability, inflammation, and cell survival block (63, 64). EC metabolic dysfunction is a key feature associated with a variety of pathological settings, including sepsis, diabetes, atherosclerosis, and ARDS, in which defective endothelial reprogramming leads to aggravated endothelial injury and vascular damage. During sepsis, an LPS challenge in ECs leads to increased glycolysis, mitochondrial dysfunction, and ROS generation, resulting in barrier disruption and inflammation (65). Also, hyperglycemia in diabetes damages the metabolism of the endothelium, causing oxidative stress and inflammation that affects flow-mediated dilation (66, 67). These metabolic changes frequently include augmented glycolytic flux and diminished mitochondrial OXPHOS, leading to cellular energy dyshomeostasis, as well as to the activation of proinflammatory signaling pathways (68). These functional changes are modulated by specific metabolic enzymes and pathways. The HIF-1α pathway also regulates endothelial metabolism by upregulating glycolysis and downregulating mitochondrial respiration, thus altering vascular tone and inflammatory reactions (69). Context-dependent endothelial metabolism modulation by the peroxisome proliferator-activated receptor beta/delta agonist GW0742: implications for neovascularization (70). The peroxisome proliferator-activated receptor β/δ (PPARβ/δ) agonist GW0742 either promotes glycolysis or FAO, depending on the context. In addition, lipid metabolism of the endothelium, such as in sphingolipid pathways, plays a role in vascular inflammation and barrier integrity, where imbalanced ceramide and sphingosine-1-phosphate signaling also participates in endothelial dysfunction (71).

**Figure 3:**
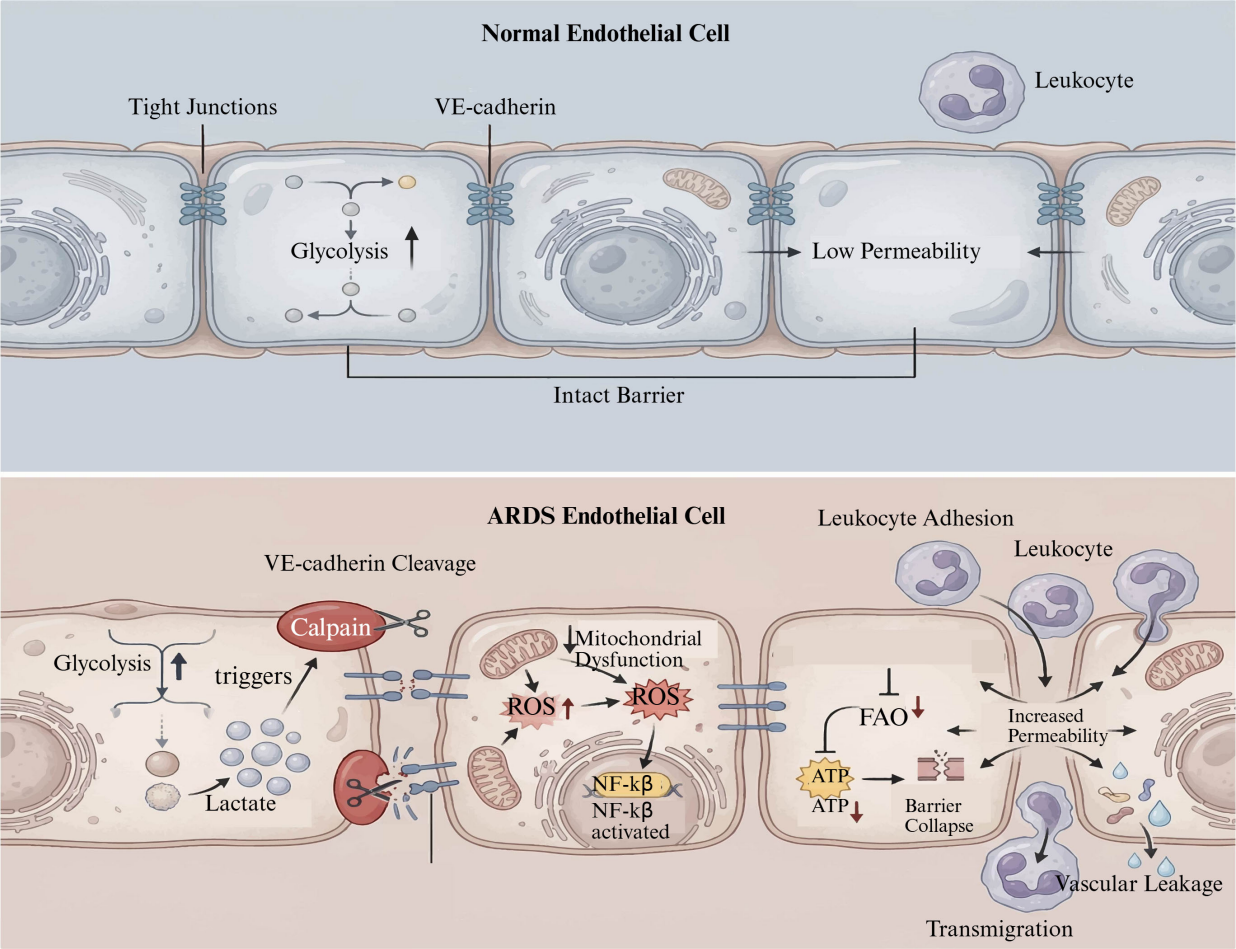
Endothelial cell metabolic abnormalities and barrier dysfunction (79). [Created with BioRender.com (11)].

Endothelial metabolites also act in a paracrine fashion to influence neighboring cells and systemic metabolism. Early studies have demonstrated that endothelial-derived lactate acts as a metabolic fuel for pericytes, connecting endothelial metabolism to vascular stability and blood-brain barrier integrity (**Figure 3**) (72). Metabolic reprogramming of ECs contributes to abnormal vessel expansion and leakage, as in pathologic angiogenesis such as tumors or retinal neovascular diseases, emphasizing metabolism as a therapeutic target (73, 74).

Metabolic enzymes, such as monoglyceride lipase (MGLL), have been linked to endothelial senescence and vascular stiffness, with pharmacological inhibition of MGLL by terazosin reducing palmitic acid-induced endothelial dysfunction (75). In addition, proinflammatory cytokines induce cytochrome P450 enzymes in ECs, modulating local drug metabolism and vascular inflammation (76). These findings reveal the complexity of the intimate network between endothelial metabolism, inflammatory signaling, and vascular physiology. Ultimately, the metabolic status of ECs serves as a dynamic controller of their phenotypic behavior. Modifications in cellular metabolism, including glycolysis, FAO, mitochondrial homeostasis, and lipid metabolism, control ECs permeability and inflammatory signaling. Impairment of these metabolic pathways is a central mediator of endothelial dysfunction in various pathologies, including sepsis, diabetes, atherosclerosis, and cancer. Unraveling the molecular circuits that orchestrate endothelial metabolism to rescue vascular.

### 2.3 Immunometabolic reprogramming of macrophages and their functional changes in sepsis-induced ARDS

#### 2.3.1 Sepsis-induced macrophage metabolic reprogramming patterns

Sepsis impacts macrophage metabolism profoundly, initiating a metabolic reprogramming that is crucial for their immune function and inflammatory phenotype. A hallmark of this reprogramming is the promotion of glycolysis in response to PAMP, including LPS. LPS-activated macrophages, via toll-like receptor 4 (TLR4), induce signaling pathways that upregulate enzymes in the glycolysis pathway and enhance glucose uptake. This Warburg-like metabolic reprogramming, leading to increased glycolysis and suppressed OXPHOS, supports an increase in ATP production and provides the biosynthetic capacity required for proinflammatory mediator generation. 6-Phosphofructokinase muscle type (PFKM), for example, is a rate-limiting enzyme in glycolysis and is highly expressed in macrophages undergoing sepsis, thereby increasing the generation of cytokines such as IL-1β, IL-6, and TNF-α. Recombinant thrombomodulin (rTM) was found to suppress PFKM expression through the HIF-1α/methyltransferase-like protein 3 (METTL3) axis, which blocks glycolysis and alleviates inflammation in sepsis models (77). The sphingosine kinase 1(SphK1)/sphingosine-1-phosphate receptor 3 (S1PR3) axis is also reported to promote glycolysis and M1 macrophage polarization in sepsis, whereas pharmacological blockade of SphK1 downregulates critical glycolytic enzymes and attenuates multiorgan injury (78).

The glycolytic intermediates are also accumulated, and a change in mitochondrial function as well. LPS exposure results in mitochondrial damage, associated with a reduction in membrane potential and an elevation in ROS content, which further escalates the inflammatory response (**Figure 3**) (58). The communication between glycolysis and mitochondrial metabolism is crucial, as mitochondrially produced ROS during reprogramming may activate inflammasomes such as NLRP3, promoting inflammation (80). Metabolic regulators, such as zinc fingers and homeoboxes 2 (Zhx2)-Zhx3, also mediated glycolysis by upregulating 6-phosphofructo-2-kinase/fructose-2,6-biphosphatase 3 (Pfkfb3) and accelerated the development of sepsis, particularly with respect to macrophage metabolism (81). In addition, ligands such as interleukin-1 receptor type II (IL-1R2) can also neutralize glycolysis-induced pyroptosis by sponging a glycolytic enzyme and exerting host-protective effects on inflammatory bowel disease (82). Mechanistically, at a more downstream level in the glycolytic pathway, macrophage polarization is believed to be defined by different metabolic signatures; thus, M1 Proinflammatory activation mainly depends on glycolysis as an energy source. Whereas M2 anti-inflammatory activity tends towards FAO and oxidative respiration. Esculetin is found to induce the repolarization of macrophages towards M1 and inhibit M2 by inhibiting glycolysis in M1 but inducing FAO in M2, protecting septic ALI (**Figure 2**) (83). Moreover, endothelial-driven factors such as C-C motif chemokine 7(CCL7) may contribute to macrophage metabolic reprogramming and M1 polarization through signal transducers and activators of transcription 1(STAT1) succinylation-mediated reprogramming, linking metabolic changes with epigenetic dysfunction and potentially affecting immune responses and inflammatory pathways during septic ALI (84). These results indicate that sepsis-induced metabolic reprogramming of macrophages involves increased glycolysis stimulated by pathogens, which in turn utilizes the metabolites and key enzymes to coordinate the inflammatory response. Targeting such metabolic processes may represent a significant therapeutic potential for regulating macrophage properties and limiting sepsis-induced tissue injury (**Figure 2**).

#### 2.3.2 Impact of metabolic reprogramming on macrophage polarization

Macrophage polarization towards proinflammatory (M1) or anti-inflammatory (M2) phenotypes is tightly modulated through metabolic reprogramming, which controls their functional and immune plasticity. Macrophage metabolism is driven in a direction that directs polarization state from the level of metabolic pathways, involving glycolysis, TCA cycle, FAO, and amino acids. M1 macrophages, classically activated by IFN-γ and LPS among others, predominantly rely on aerobic glycolysis to produce metabolic intermediates that could be used for rapid ATP generation and biosynthetic precursors to carry out proinflammatory functions such as production of ROS and cytokine secretion (79, 85). This metabolic reprogramming is responsible for the production of proinflammatory cytokines, which help clear the pathogen but can also lead to tissue injury if unrestrained (86). On the other hand, M2 macrophages induced by IL-4 or IL-13 also require OXPHOS and FAO for their anti-inflammatory properties, whereas these markers are less prominent among M(IL-10) (79, 85). This metabolic homeostasis is crucial for maintaining immune balance and resolving immune responses. Metabolic reprogramming not only influences macrophage polarization but also affects their efficiency in phagocytosis and cytokine production. The accumulation of TCA cycle intermediates, such as succinate, in M1 macrophages causes the stabilization of HIF-1α, which in turn enhances glycolytic gene expression and proinflammatory cytokine production, including IL-1β (87). On the other hand, metabolites including itaconate and α-ketoglutarate have been reported to inhibit inflammation and promote M2 polarization by regulating epigenetic and signaling cascades (85). In addition, amino acid metabolism has been shown to illustrate the metabolic polarization defining M1 and M2 macrophage function, in particular arginine metabolism through inducible nitric oxide synthase (iNOS) in M1, and arginase-1 (ARG1) expression in M2 macrophages (88). The same metabolic enzymes can also influence the production of NO by macrophages, as well as polyamine synthesis, thereby regulating macrophage-mediated immune reactions. Recent studies have highlighted the significance of lipid metabolism in macrophage polarization. TAMs, frequently characterized by an M2-like status, undergo metabolic reprogramming to contribute to tumor promotion by increasing lipid intake and fatty acid synthesis (89, 90). Solute carrier family 3 member 2 (SLC3A2) in lung cancer cells affects macrophage polarization by arachidonic acid metabolism so that M2-type pro-tumor suppressive macrophages are increased (91). Likewise, cholesterol metabolism also regulates macrophage polarization in atherosclerosis, and elevated cholesterol biosynthesis promotes M2 polarization as well as inhibits anti-tumor immunity (92). These data demonstrate the complexity of metabolic instructive cues in regulating macrophage phenotypes within various microenvironment niches.

Targeting macrophage metabolism is of therapeutic interest. Pharmacological inhibition of metabolic enzymes or pathways alters macrophage polarization and ameliorates disease. Inhibition of Pim2 kinase attenuates glycolysis/M1 polarization in inflammatory arthritis, thereby ameliorating the disease (93). There are also some natural products, including quercitrin and songorine, that were reported as M2 macrophage polarization inducers, possibly mediated by enhancing mitochondrial OXPHOS but inhibiting glycolysis and then reducing inflammation in MI and osteoarthritis mouse models (94). In addition, reprogramming in nicotinamide adenine dinucleotide (NAD+) metabolism contributes to macrophage polarization and immunosuppression in cancer and radiotherapy (95). These interventions demonstrate that the immunometabolism can be specifically addressed to influence macrophage function and the inflammatory process.

Sumoylation (SUMOylation), as a reversible post-translational modification of proteins, participates in regulating the activity of the nuclear receptor peroxisome proliferator-activated receptor γ (PPARγ), the synthesis of ubiquinol-ytochrome C reductase core protein 1(Uqcrc1), and signaling pathways such as mammalian target of rapamycin complex 1(mTORC1)/p70S6K, which are involved in macrophage metabolic regulation and polarization (95). In ARDS, SUMOylation modification may affect the stability and activity of HIF-1α, thereby regulating the expression of glycolysis-related enzymes, reducing the secretion of proinflammatory cytokines, and alleviating the inflammatory response. growth differentiation factor 15(GDF15), by inhibiting the HIF-1α-mediated glycolysis pathway, reduces the expression of inflammatory factors, alleviating ECs inflammation and barrier damage (84). Key enzymes and signaling molecules regulated by SUMOylation, like the SphK1/S1PR3 axis, are involved in controlling macrophage glycolysis activity and promoting proinflammatory M1 polarization; inhibiting this axis helps promote M2 polarization, alleviating inflammation and tissue damage (96). SUMOylation may regulate the stability of the NLRP3 protein or its interaction with the adaptor protein ASC, suppressing inflammasome overactivation through NF-κB and reducing inflammatory damage. Glycolysis inhibitor lonidamine and metabolic regulator itaconate derivatives can effectively suppress cytokine storms and restore immune homeostasis, providing potential intervention targets for SUMOylation-regulated cytokine secretion imbalance (78).

Metabolic reprogramming acts as a major influencer in shaping macrophage polarization and controls their proinflammatory or anti-inflammatory manifestations via specific metabolic pathways and intermediates. This metabolic regulation also governs phagocytosis and cytokine production, which in turn affects immunity as well as tissue integrity. Elucidation of the complexities of how metabolism integrates with macrophage function presents opportunities for therapeutic approaches for inflammatory diseases, cancer, and tissue repair by targeting macrophage polarization with metabolic interventions.

#### 2.3.3 Relationship between macrophage metabolism and pyroptosis

**Figure 4:**
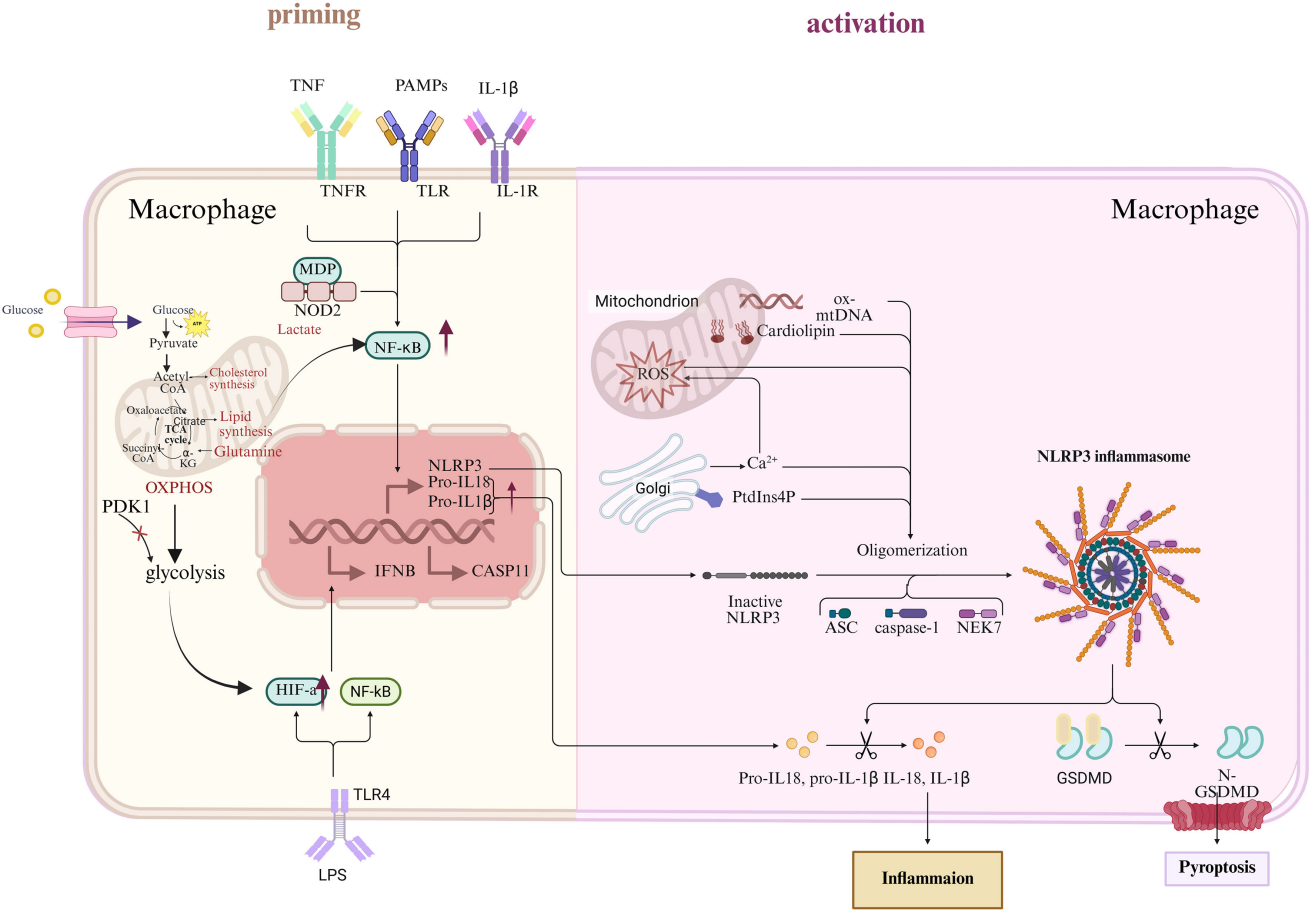
Macrophage metabolism and pyroptosis. [Created with BioRender.com (11)].

Macrophage metabolic states critically regulate pyroptosis signaling pathways, thereby influencing the inflammatory cascade that characterizes ARDS in sepsis. Gasdermin D (GSDMD) is the main substrate of caspase-1, and it can be cleaved by specific immune cells to mediate organismal defense mechanisms through its induction of a proinflammatory response during pyroptosis. This cascade leads to the generation of proinflammatory cytokines, including interleukin (IL)-1β and IL-18, that further aggravate the local inflammatory environment. The reprogramming of metabolism in macrophages, particularly the switch from OXPHOS to glycolysis, is a key regulator of pyroptosis. LPS-activated macrophages grown in high glucose adopt increased glycolysis, a process that increases the presence of HIF-1α, a transcriptional factor that upregulates IL-1β expression and stimulates inflammasome activity temporarily. This metabolic state promotes pyroptosis, as indicated by caspase-1 activity and GSDMD cleavage being up-regulated (97). Mechanical perturbation can also disrupt macrophage energy metabolism and induce dysregulation of lactate dehydrogenase A (LDHA) and pyruvate dehydrogenase (PDH), as well as mitochondrial damage, followed by caspase-1-dependent pyroptosis and sterile inflammation (98). Inhibitors against the key metabolic regulators, like pyruvate dehydrogenase kinase 1 (PDK1), can modulate glucose imbalance and inhibit pyroptosis, suggesting a tight link between metabolism reprogramming and programmed cell death paths. Glycolysis reprogramming also regulates macrophage pyroptosis through the action of metabolic signaling molecules, such as AMPK/SIRT1/NF-κB, in a mouse model of periodontitis. Inhibition of glycolysis by 2-deoxy-D-glucose (2-DG) suppresses macrophage pyroptosis, thereby ameliorating inflammatory bone resorption and suggesting that metabolic activity determines the fate choice between life and death (99). Likewise, corneal epithelial hyperosmolarity-engaged glycolytic reprogramming can drive macrophage pyroptosis and inflammation that can be abrogated by blocking glycolysis. By doing so, these results combine to demonstrate that the metabolic states of macrophages are upstream regulators of inflammasome activation and pyroptosis. Additionally, the production of cytokines during pyroptosis exacerbates the prostate-associated inflammatory milieu typical for ARDS. Pyroptotic macrophages secrete IL-1β and IL-18 through GSDMD-derived membrane pores to attract and activate more immune cells that continue the inflammatory cascade. Hepatocyte pyroptosis-mediated macrophage re-metabolism to a proinflammatory M1 phenotype is involved in acute liver injury, leading to tissue damage. In metabolic disorders, including but not limited to osteoporosis and atherosclerosis, macrophages undergoing pyroptosis elicit pathological inflammation and tissue remodeling through the cytokines they release, thereby reshaping the local immune environment (100). Indeed, ROS produced during metabolic stress function as signaling molecules that trigger the activation of NLRP3 inflammasome, connecting oxidative metabolism to pyroptotic form of cell death (101). Drugs that target those metabolic controllers or inflammasome elements, like methylophiopogonanone A (MO-A), which inhibits ROS generation and NLRP3 activation, prominently restrain macrophage pyroptosis and inflammation (102) (**Figure 4**).

Further evidence of the crosstalk between metabolism and pyroptosis in sepsis is demonstrated by the interaction between IL-1R2 and enolase 1 (ENO1), in which IL-1R2 inhibits ENO1-induced glycolysis, thereby attenuating GSDMD-dependent pyroptosis and inflammation (82). On the contrary, metabolic stressors such as palmitic acid initiate macrophage pyroptosis through c-Fos/dual-specificity phosphatase 1(Dusp-1) signaling activation that connects lipid metabolism with inflammatory cell death and endothelial dysfunction (103). These data indicate that metabolic reprogramming not only controls pyroptosis initiation but also conditions the downstream effects of the inflammasome, thereby worsening ARDS severity. Overall, the findings from these studies provide a better understanding of an intricate and bidirectional connection between macrophage metabolism and pyroptosis. Metabolic changes towards glycolysis and mitochondrial impairment act as initiators of inflammasome activation and pyroptotic cell death, where pyroptosis-related cytokines enhance inflammation and aggravate metabolic disarray. This crosstalk forms a vicious circle that enhances lung injury in sepsis-induced ARDS. Targeting metabolic enzymes, signaling pathways, or inflammasome components may provide potential therapeutic strategies for the regulation of macrophage pyroptosis and hyperinflammation in ARDS progression.

#### 2.3.4 Epigenetics in the immunometabolic reprogramming of macrophages in sepsis-induced ARDS

Epigenetic mechanisms fine-tune cell interactions by regulating the expression of intercellular signaling molecules. Histone demethylase UTX regulates miRNA expression in ECs, thereby affecting macrophage polarization and promoting the alleviation of inflammation (104). miRNAs not only regulate the metabolism of immune cells but also participate in the regulation of immune function and barrier integrity of ECs. Their expression and function are jointly influenced by DNA methylation and histone modification (105). In macrophages, changes in histone acetylation levels are directly correlated with their metabolic state and polarization direction. High acetylation levels help maintain the OXPHOS metabolic state of macrophages and promote M2 polarization; whereas deacetylation tends to support glycolysis metabolism and promote M1 polarization. This coupled regulation mechanism of metabolism and epigenetics is crucial for the flexible regulation of macrophage function. The ACLY enzyme provides substrates for histone acetylation by generating acetyl-CoA, linking lipid metabolism and histone acetylation, further affecting the expression of immune genes and the functional state of macrophages. In ARDS, HDAC inhibitors are expected to become therapeutic strategies by regulating the epigenetic state of inflammation-related genes and reducing the excessive activation of inflammatory cells (106). Various miRNAs regulate the balance of macrophages in metabolic processes such as glycolysis and FAO by targeting metabolic enzymes and signaling pathways, thereby affecting their immune phenotype and function. MiR-155 exhibits a dual regulatory role in macrophage polarization: its upregulation promotes glycolysis metabolism enhancement, supporting the formation of M1 proinflammatory phenotype; whereas miR-146a inhibits excessive inflammatory response through a negative feedback mechanism, while regulating metabolic pathways to promote the transition of macrophages towards anti-inflammatory M2 phenotype (107).

### 2.4 The role and mechanisms of ECs metabolic reprogramming in sepsis-induced ARDS

#### 2.4.1 ECs metabolic changes and their impact on vascular permeability

ECs undergo metabolic reprogramming, which significantly impacts vascular permeability —a central pathogenic mechanism in sepsis-associated ARDS and other inflammatory vascular disorders. A major metabolic reprogramming is the activation of glycolysis, which leads to endothelial activation and barrier dysfunction (**Figure 3**). It has been reported that prolonged stimulation of ECs by catecholamines, like adrenaline and noradrenaline, induced glucose consumption and aerobic respiration processes, which reflects a switch in metabolism pathways, probably favoring endothelial activation and oxidative stress signaling. This glycolytic increase is consistent with observations in disease states, e.g., sepsis and ARDS, where ECs demonstrate enhanced lactate production. High lactate activates calpain proteases, via ERK, to disrupt VE-cadherin complexes, resulting in VE-cadherin cleavage and increased endocytosis, causing endothelial hyperpermeability (108). In addition, the exposure of cerebral ECs to fibrillar tau species associated with neurovascular disease caused a Warburg-like reprogramming towards glycolysis, leading to increased inflammatory polarization and dysregulation of tight junction integrity, further supporting that their glycolytic rewiring affects the endothelial barrier. Metabolic products also modulate tight junction proteins that are critical for endothelial barrier integrity. Ethanol increases the epigenetic hypermethylation of the ZO-1 promoter in endothelial permeability through a DNA methyltransferase-3a (DNMT3a)-dependent manner that is mediated by metabolic stress and mitochondrial dysfunction. Donors of hydrogen sulfide (H_2_S) reduce this hypermethylation and are implicated in maintaining expression of tight junction proteins and the integrity of the vasculature (109). Likewise, under diabetic conditions, PACS2-mediated dysregulation of FAO is responsible for endothelial barrier breakdown. Exposure to high-glucose and palmitic acid decreases FAO, ATP formation, VE-cadherin internalization, and increases permeability response that can be reversed by pretreatment with PACS2 inhibitor, plus replenishing the level of FAO (110). Taken together, our collective data emphasize the relationship between metabolic waste products and changed substrate use with tight junction components and endothelial barrier function.

The endothelial glycocalyx, primarily composed of glycosaminoglycans, with hyaluronan playing a key role, is another major mechanistic determinant of vascular permeability. Its synthesis and turnover are closely integrated with glucose metabolism in the endothelium. Disruption of glycocalyx integrity in diabetes and inflammation leads to increased vascular permeability and inflammation. The presence of sugar substrates for glycocalyx synthesis also links metabolic derangements to loss of structural barrier. Re-wiring of lipid metabolism, through metabolic reprogramming, also influences the features of the endothelial barrier. Suppression of angiopoietin-like 4 (ANGPTL4) expression in ECs is able to induce metabolic reprogramming toward increased fatty acid utilization and oxidation and decreases pathologic neovascularization as well as vascular permeability (111), suggesting that lipid metabolism serves a determinant role for the regulation of endothelium barrier integrity. Calcium signaling and phosphoinositide metabolism are also metabolic mediators of endothelial permeability. PLCβ2 increases VEGF-stimulated permeability by regulating intracellular calcium and phosphatidylinositol bisphosphate 4,5 (PIP2) in conjunction with tight junctions and cytoskeletal reorganization. Suppression of VEGF-induced permeability by deletion of PLCβ2 and disease outcomes in lung injury models suggests that metabolic signaling pathways are a therapeutic target to regulate endothelial barrier function (112). Taken together, these studies reveal that there is a multifaceted relationship between endothelial metabolic reprogramming-the increase in glycolysis and remodeling of lipid and amino acid metabolism-and the control of tight junction proteins and vascular permeability. Through these metabolic intermediates and signaling pathways, ECs activation, tight junction integrity, and cytoskeletal arrangement are regulated, leading to a disrupted vascular barrier in sepsis-induced ARDS along with other inflammatory vascular diseases. Novel therapeutic interventions, including inhibition of glycolysis, restoration of FAO, epigenetic regulation, and targeting metabolic enzymes, offer hope to ameliorate vascular barrier function and minimize organ injury in critical illness.

#### 2.4.2 ECs metabolic abnormalities and activation of inflammatory signaling pathways

ECs play a pivotal role in maintaining vascular homeostasis, and their dysfunction is a hallmark of various inflammatory and metabolic diseases, including sepsis-associated ARDS and atherosclerosis. Metabolic reprogramming in ECs, including glucose/lipid metabolism shift, mitochondrial dysfunction, and oxidative stress, is closely associated with such regulatory signaling pathways as NF-κB and HIF-1α. These pathways coordinate the transcriptional control of proinflammatory mediators, adhesion molecules, and chemokines, which promote endothelial inflammation and barrier function.

Indeed, one key way in which metabolic reprogramming potently activates inflammatory signaling is through the generation of ROS resulting from dysfunctional mitochondria. Elevated mitochondrial superoxide production in senescent or stressed ECs results in oxidative damage and NF-κB activation, culminating in the release of cytokines and adhesion molecules that promote leukocyte recruitment and vascular inflammation (113). During SARS-CoV-2 infection, mitochondrial DNA (mtDNA), upon release from the damaged organelles, triggers toll-like receptor 9 (TLR9) activity, fostering the NF-κB pathway and proinflammatory cytokines generation that contribute to endothelial dysfunction (114). In addition, during metabolic diseases such as obesity and diabetes, ECs are subjected to the proinflammatory effects of a high concentration of saturated fatty acids through Toll-like receptor (TLR)-mediated pathways and long-chain acyl-CoA synthetase-1 (ACSL1)-dependent mechanisms (115), thereby once again linking metabolic insult with inflammation.

SUMOylation may play a role in regulating the GDF15 signaling pathway, thereby enhancing its protective effects, stabilizing the ECs barrier, and reducing leukocyte adhesion and alveolar damage. The chemokine CCL7 secreted by ECs activates STAT1 through the CCR1 receptor, inducing lysine succinylation, which promotes the transcription of glycolytic genes and drives M1 polarization and inflammatory cascade responses. SUMOylation modification can interfere with this process, inhibiting STAT1 activation, preventing metabolic reprogramming, and reducing proinflammatory macrophage polarization (116).

Hypoxia and altered oxygen sensing also contribute to EC metabolic reprogramming and inflammation. Stabilization of HIF-1α in hypoxic or inflammatory conditions results in a shift towards glycolytic metabolism and induction of inflammatory genes,10 maintaining endothelial activation. This metabolic adaptation is beneficial to the elevated energy demands of activated ECs, yet amplifies oxidative stress and vascular damage. Autophagy-related 7 homolog (ATG7), an endothelial autophagy-related gene, has also been found to regulate EC metabolism and inflammation. Its deletion is protective against high-fat diet-induced obesity and vascular rarefaction through the maintenance of endothelial function and diminished lipotoxicity (117). The oxidative stress and metabolic dysregulation are synergistic in ECs, resulting in a vicious cycle of endothelial injury. ROS generation is upregulated, interfering with calcium signaling pathways that are essential to endothelial barrier integrity and vasorelaxation, while antioxidant defenses protecting against oxidative damage are compromised. Activation of the protein kinase C (PKC) isoforms under hyperglycemic and oxidative conditions increases ongoing inflammatory signaling and alters endothelial permeability (118).

Interventions in these metabolic and inflammatory pathways are thus becoming increasingly promising. The transcriptional and immunoresponsive regulator (TCM) Typhae Pollen extract attenuates endothelial senescence and inflammation by suppressing NF-κB and redox pathways (119). Pharmacological approaches, including treatment with dapagliflozin, suppress endothelial inflammation and oxidative stress by modulating the PI3K/AKT pathway, which favors normal vascular function in models of metabolic diseases (120). In addition, there is less oxidative stress and inflammation when the endothelial mineralocorticoid receptor (MR) or epithelial sodium channel (ENaC) signaling is modulated, underscoring regulatory ion channels as important components of metabolic endothelial dysfunction (121, 122).

Oxidative stress and metabolic dysfunction interact in a feed-forward manner that maintains inflammation and vascular disease. Mechanistic insight offers the opportunity to design targeted therapies to reconstitute endothelial homeostasis in sepsis-induced ARDS and other metaboinflammatory vascular diseases.

#### 2.4.3 Metabolic regulation on ECs apoptosis and repair

The metabolic status of ECs is crucial for maintaining a balance between survival and apoptosis, thereby influencing vascular homeostasis and repair. ECs have a high demand for energy and biosynthetic processes, which are primarily fulfilled through various metabolic pathways, including glycolysis, FAO, and mitochondrial respiration (**Figure 3**). Under pathophysiological conditions, dysregulation of these metabolic features contributes to endothelial dysfunction characterized by enhanced apoptosis and reduced regeneration potential. Hyperglycemia-associated oxidative stress evokes mitochondrial damage and EC apoptosis through pathways of ROS accumulation and ATP production deficiency (123). Likewise, oxidation of low-density lipoprotein (oxLDL) and metabolic stressors also lead to mitochondrial damage and intrinsic apoptosis through reduced expression of key mitochondrial proteins. Transmembrane protein 70 (TMEM70) is involved in the assembly of ATP synthase (124). The activity of HIF-1α is also regulated by miRNAs, which mediate the survival of ECs in ischemic conditions, thereby linking metabolic sensing to apoptotic pathways (125). In addition, the alterations in metabolism, including chemical reprogramming from OXPHOS to glycolysis as seen in pulmonary arterial hypertension (PAH), lead to apoptosis resistance and pathological ECs proliferation (126). Master regulators of EC metabolism and function, such as exemplary signaling intermediary molecules like interferon regulatory factor 1 (IRF-1) and forkhead box O3a (FOXO3a), are at the crossroads of metabolic and inflammatory signals, mediating EC apoptosis and inflammation (127). Metabolic pathway-targeting agents, such as rosuvastatin, cannabidiol, and galangin, have been shown to contribute to protection against endoplasmic reticulum (ER) stress-induced apoptosis, along with autophagy-dependent survival, in ECs (128–130). Paracrine factors, like insulin-like growth factor 1(IGF-1) released by mesenchymal stem cells, can also induce the activation of the PI3K/Akt/mTOR signaling pathway in lymphatic endothelial progenitor cells, promoting proliferation and inhibiting apoptosis (131). Collectively, these findings highlight that ECs destiny is strongly regulated by metabolic conditionality, as changes in energy supply/redox balance, as well as nutrient utilization, predominate the pro-apoptotic/survival decision. Knowledge of these metabolic regulatory mechanisms may suggest therapeutic targets that may preserve endothelial integrity and blood vessel homeostasis in diseases like sepsis-related ARDS, vascular complications associated with diabetes, and atherosclerosis. Metabolic reprogramming is also crucial for promoting endothelial repair and restoring vascular homeostasis after injury. ECs possess an extraordinary degree of metabolic plasticity, enabling them to facilitate reparative functions such as proliferation, migration, and angiogenesis. Hypoxia-induced activation of the HIF pathway is such an example where, under low oxygen conditions, ECs increase glycolysis to meet energy demands and support angiogenesis even in the presence of sustained hypoxic conditions (132, 133). The glycolytic dependence of ECs is required for survival and function during ischemic injury and tissue regeneration (134).

Additionally, metabolic intermediates such as lactate serve as signaling molecules for the repair of endothelial and neuronal cells, as demonstrated by the use of biomaterial scaffolds, where the release of lactate induces nerve regeneration through ATP production and angiogenesis (135). Fatty acid metabolism and protein palmitoylation also influence endothelial function and repair by regulating NO synthesis, inflammatory signaling, and membrane dynamics (136). Furthermore, endogenous gasotransmitters, including sulfur dioxide (SO_2_), regulate ECs apoptosis and contribute to vascular homeostasis (137, 138). Epigenetic regulation through metabolic pathways further regulates endothelial gene expression to ensure reparative processes (139). Endothelial-immune cell crosstalk of metabolic and angiocrine factors is instrumental to the resolution of inflammation and vascular remodeling (140). Therapeutic approaches targeting metabolism to alleviate dysfunction or drive repair are emerging, with PPAR targeting GDF11 being an example, leading to reduced senescent ECs and improved mitochondrial dynamics through chloride channels (141). Altogether, these remarks indicate that metabolic control not only regulates ECs survival but is also critically involved in promoting repair and restoration of vascular homeostasis. Metabolic pathway intervention appears to be an attractive therapeutic option, improving endothelial repair and treating vascular dysfunction in sepsis-induced ARDS and related syndromes.

#### 2.4.4 Epigenetics in the immunometabolic reprogramming of EC in sepsis-induced ARDS

Histone modification, a crucial form of epigenetic regulation, plays a pivotal role in regulating the function of ECs and inflammatory responses. Acetylation and methylation modifications of histones can directly affect the expression of inflammatory genes in ECs, thereby controlling the expression levels of cell adhesion molecules such as ICAM-1 and vascular cell adhesion molecule-1 (VCAM-1), influencing the adhesion process between leukocytes and ECs, promoting the infiltration of inflammatory cells, and exacerbating local inflammatory responses. Under high glucose conditions, the level of histone H3K27 trimethylation (H3K27me3) increases in ECs, leading to the suppression of the expression of anti-inflammatory transcription factors such as kruppel like factor 2 (KLF2) and KLF4, prompting ECs to exhibit an inflammatory activation state (142). Furthermore, histone methyltransferase set domain bifurcated 1(SETDB1) is upregulated under hypoxia stimulation through a HIF2α-dependent mechanism, promoting apoptosis, senescence, and endothelial-mesenchymal transition (EndoMT) in pulmonary microvascular ECs, resulting in endothelial dysfunction and pulmonary vascular remodeling. This also provides a new perspective on the pathogenesis of pulmonary arterial hypertension (116). MicroRNAs (miRNAs) can influence the functional status of ECs by modulating their response to external stimuli, including oxidative stress, inflammatory factors, and metabolic imbalances. Specific miRNAs, such as miR-148a-3p, in ECs of patients with ARDS regulate cytoskeleton remodeling by targeting the ROCK1 signaling pathway, protect the endothelial barrier function, reduce cell permeability and inflammatory response, thereby alleviating lung injury (143). miR-126 and miR-21 are miRNAs abundantly expressed in ECs and play essential roles. miR-126 plays a central role in maintaining vascular endothelial homeostasis and promoting vascular repair, regulating angiogenesis, ECs migration, and proliferation, and inhibiting abnormal increases in vascular permeability (144).

### 2.5 Interaction mechanisms between macrophages and ECs and immune metabolic regulation

#### 2.5.1 Cytokine- and chemokine-mediated interactions

In sepsis-associated ARDS, the crosstalk of macrophages and ECs is highly regulated by cytokines and chemokines that play a key role in mediating immune and vascular responses. Activated macrophages release proinflammatory cytokines, which are known to be activators of ECs. These cytokines lead to ECs activation, characterized by the upregulation of adhesion molecules, aberrant vascular permeability, and the additional release of inflammatory mediators. This activation induces not only the adhesion and transmigration of leukocytes but also the aggravation of endothelial dysfunction, a characteristic feature of ARDS pathogenesis. TNF-α and IL-1β are therefore considered central mediators, acting as the initial signal that enhances endothelial inflammation to mediate the onset of alveolar-capillary barrier disruption, characterized by the development of ARDS. Conversely, activated ECs secrete a panel of chemokines that attract circulating macrophages, thus promoting more complex signals involved in inflammation. Endothelial expression of chemokines, in response to inflammatory stimuli is capable of recruiting monocytes/macrophages. This chemokine gradient provides a mechanism for macrophages in the lung, circulating through the microvasculature, to be recruited to and/or retained at sites of endothelial injury and inflammation. Macrophage accumulation is exacerbated by ongoing cytokine secretion and metabolic reprogramming that preferentially promotes macrophages towards an activated state. This mutual relationship results in a feed-forward loop, where macrophage-released cytokines activate ECs, which in turn produce chemokines to recruit additional macrophages, thereby intensifying the lung injury.

Moreover, these interactions rely on immunometabolic reprogramming in addition to the metabolic features of macrophages and impact their cytokine secretory profile and migration properties. An energy metabolism regulator, like the sedoheptulose kinase, regulates macrophage activation and chemokine receptor expression, which drives its response to endothelial-derived chemokines and its migratory capacity toward inflamed tissues. Changes in metabolic pathways, including glycolysis and the pentose phosphate pathway (PPP), are not only necessary for generating energy to support macrophage activation but also for integrating redox balance and biosynthetic processes required for cytokine synthesis. This metabolic checkpoint is critical for sustaining a proinflammatory cross-talk between macrophages and ECs in sepsis-induced ARDS. Taken together, the cytokine/chemokine side-by-side interaction between macrophages and ECs may be a central mechanism cascading inflammation and endothelial impairment in sepsis-induced ARDS. Targeting these signaling pathways, as well as their associated immunometabolic machineries, may provide attractive therapeutic opportunities for manipulation of macrophage/endothelial crosstalk, which governs a lung-protective condition and potential clinical recovery. The complex interplay of these interactions within the context of the gut-lung axis serves to highlight even more the rationale for an integrated approach targeting systemic inflammation and local pulmonary effects.

#### 2.5.2 Role of metabolic products in intercellular signal transmission

Metabolites, including lactate and succinate, are currently in the spotlight not only as metabolic intermediates but rather as dynamic modulators of cell activities and intercellular communication. Lactate has not only transitioned from a metabolic byproduct created downstream of glycolysis to a recognized signaling molecule capable of reprogramming cell fate and immune function. During early embryogenesis, lactate production is specifically upregulated in primitive endoderm (PrE) cells, where it cooperates with fibroblast growth factor (FGF) signaling from epiblast precursor cells to form an intercellular positive feedback loop that drives lineage specification. Lactate-derived signaling may also be linked to epigenetic regulation through histone lactylation, establishing a connection between metabolic reprogramming and gene expression and cell differentiation (145). Within the microenvironment of tumors, lactate accumulation mediates the lactylation modification of proteins, like MOESIN, by TGFβ-related signals and immunosuppressive activity, thereby stabilizing the function of regulatory T cells (146). These results reinforce the complexity of lactate as both an energy source and a paracrine signaling molecule that modulates cell crosstalk.

Likewise, succinate - another Krebs cycle intermediate - provides a prime example for the dual function of metabolites as intracellular metabolic substrates and extracellular signaling molecules. Succinate secretion into the microenvironment can act on the cell surface receptors of adjacent cells, influencing inflammatory responses and cell–cell communication. The α-keto acid dehydrogenase complexes, mainly the α-ketoglutarate dehydrogenase in this case, are regulators of succinate and other metabolite availability and act as intracellular and extracellular signaling scaffolds by a redox-dependent fashion. These enzyme complexes regulate mitochondrial metabolism and epigenetic reprogramming by controlling the fluxes of metabolites and signaling through ROS, which in turn mediate intercellular interactions (147).

The signaling metabolites are also particularly important for either inducing or inhibiting cellular interactions promoting tumor growth. Metabolites can control signaling cascades by binding to and either enforcing or blocking specific receptors, or by manipulating proteins through post-translational modifications (PTMs), including metabolite-mediated acetylation, lactylation, or succinylation. Additional examples of histone modifications that may adjust gene expression and inevitably cell function are observed in lactate at “lactylation,” which modifies chromatin accessibility to shape differential programming of ICOS-L dependent intercellular communication and cell phenotypes (145). Similarly, metabolites such as fructose-1,6-bisphosphate (FBP) have influences on mitochondrial enzyme aldehyde dehydrogenase 2 (ALDH2), balancing the generation of ROS and organelle dynamics, which affect signaling to surrounding cells (148). These interactions of proteins with metabolites represent channels by which cell-level metabolic state can be directly connected to the regulation of signaling pathways that govern the diverse array of cellular behaviors and communicative functions (149, 150).

Extracellular vesicles (EV) are major players in conveying metabolites, proteins, lipids, and nucleic acid messages during intercellular communications in a broad range of normal and pathological states. EVs act as carriers of metabolites that modulate the signaling and ultimately the function of recipient cells, affecting immune responses, tissue repair, and tumor progression (151, 152). The metabolome profile of EVs may also be modulated by non-biological causes, such as exposure to microplastic particles, which act as intermediaries for intercellular communication and cell metabolism (153). These vesicle-transported metabolite exchanges present an elegant way for cells to adjust the local and systemic context intrinsically through tissues.

In addition, certain membrane channels, including connexins and pannexins, directly mediate the passage of ion and solute molecules between the neighboring cells, either affecting or modulating the strength/quality of cell-to-cell crosstalk. Connexin hemichannels and gap junctions are capable of transporting metabolites with signaling molecules to regulate tissue homeostasis and immune defense (154–156). The regulation of connexin-based communication is linked to inflammatory disease states and neoplastic growth. Therefore, maintaining appropriate intercellular signaling through these proteins is critical (157, 158). The formation of large anionic metabolite-permeable pores by CASP and pannexin-1 serves as a direct substrate for extracellular signaling (154). Emerging bioinformatics tools, like MetalinksDB and MRCLinkdb, have further expanded our understanding of metabolite-protein and metabolite-receptor binding associations, which has facilitated the prediction of metabolite-modulated intercellular signaling networks in specific biological contexts (159, 160). These databases include information that can be used to predict and analyze metabolite-based communication networks, processes often ignored in traditional protein-centric studies of cell signaling.

#### 2.5.3 Cell contact-dependent signaling and its metabolic regulation

Adhesion molecules, composed of integrins and selectins/members of the immunoglobulin (Ig) superfamily, guide cells in forming and maintaining dynamic yet tight contacts with adjacent cells. This process modulates both cellular activity and function through paracommunication with neighboring cells. Integrin-directed signaling pathways were shown to be activated by the interaction between macrophages and ECs that drives metabolic reorganization and functional repolarization in both cell types. This intimate contact is critical not only for biomechanical anchoring but also for engaging intracellular signaling pathways that mediate immune responses and vascular permeability. The crucial role of cell adhesion molecules as orchestrators of these contacts is demonstrated by the fact that inhibition of integrin binding also blocks metabolic alterations and functional consequences for target cells, such as the activation of metabolic enzymes or the synthesis of cytokines. Integrin-ligand interactions may activate mitogen-activated protein kinase (MAPK) pathways, which are linked to the induction of gene expression of metabolism/inflammation. Molecular Control of ECM Receptors between Mø and ECs. It follows that regulation of adhesion molecules expression by cytokines/chemokines might be added as an additional multistep process coupling mechanical with molecular signals for the crosstalk between immune and endothelial responses (161, 162). The metabolic status of macrophages and EC plays a pivotal role in adhesion molecule expression or functionality, with consequences on the force and quality of cell-cell contacts. Glucose concentration and mitochondrial function are metabolic signals that can control the surface expression of adhesion molecules as well as their ability to signal. Metabolic reprogramming in stromal or immune cells leads to enhanced OXPHOS and/or glycolysis and induction of higher levels of integrin and other adhesion molecule expression, which result in stabilized cell–cell interaction.

On the other hand, metabolic stress/lipid metabolism can mediate the repression of adhesion molecule expression, leading to loss of cell-cell contact and modulation of the immune response. This “crosstalk” of metabolism and adhesion is only one example of the many participations of lipid signaling molecules, which are anionic species derived from biosynthetic intermediates and also serve as signaling molecules modulating various membrane dynamics and protein localization at contact sites. Membrane contact site control of spatiotemporal PA metabolism may modulate how adhesion complexes form and function, offering a means to couple metabolic pathways with the direct regulation of cell adhesion and signaling. Furthermore, cell contact-dependent metabolic reprogramming may lead to the overexpression of enzymes, which also regulates glycolytic flux and ATP production in active cells during immune responses. Their regulation of metabolism impacts the expression and signaling capacity of adhesion molecules, which are particularly important in maintaining the balance between homeostatic immune activation/vascularity, especially in sepsis-associated ARDS when macrophage-ECs crosstalk is impaired (161–164).

Cell contact-dependent signaling between macrophages and ECs is tightly regulated by adhesion molecules whose expression and function are modulated by the metabolic state of the interacting cells. This bidirectional regulation ensures that immune and vascular responses are finely tuned according to the cellular metabolic milieu, enabling appropriate responses to inflammatory stimuli. Understanding the molecular mechanisms underlying this crosstalk, including the role of metabolic intermediates and signaling pathways such as integrin-MAPK and lipid metabolism, provides valuable insights into potential therapeutic targets for diseases characterized by immune and endothelial in ARDS. Targeting the metabolic regulation of adhesion molecules and cell contact signaling may offer novel strategies to modulate macrophage-ECs interactions and ameliorate pathological inflammation and vascular injury.

### 2.6 Gut-lung axis regulates immunometabolic reprogramming and its therapeutic potential

#### 2.6.1 Regulation of immunometabolism by gut microecology

The gut microbiota has a deep impact on the host immunometabolism through their metabolic products, such as SCFAs, bile acids, and other microbial metabolites, to influence the function of immune cells locally, including the intestinal tract, but also in various extra-intestinal compartments. SCFAs, particularly acetate, propionate, and butyrate, which are produced through bacterial fermentation of dietary fibers, are important signaling molecules that modulate both energy metabolism and immune response. They are also involved in the induction of Treg differentiation and the increased integrity of epithelial barriers, as well as reprogramming the metabolism of macrophages and dendritic cells that mediate immune homeostasis by restraining excessive activity. The modified production of these metabolites and the metabolic pathways involved in inflammation leading to ARDS may be permissive for dysbiosis or a change in enteric microbial composition from baseline. A low density of butyrate-producing bacteria and a high abundance of proinflammatory bacteria have been linked to systemic inflammation, as well as reduced immune regulation (165, 166). Indeed, the gut-lung axis captures this conversation: gut microbial metabolites traverse the circulation to the lung compartment, influencing pulmonary immune cell metabolism and function, thereby regulating susceptibility to and dynamics of ARDS. In addition, bile acids are also metabolized by the microbiome, where they act as ligands for receptors including farnesoid X receptor (FXR) and G protein-coupled bile acid receptor 1(GPBAR1), which impinges on immune cell metabolism and inflammatory pathways -providing a link between gut microbial metabolism and systemic immune regulation (167, 168). These processes fail in the setting of microbial dysbiosis and are unable to limit proinflammatory macrophage activation, endothelial dysfunction, or metabolic reprogramming that promotes lung injury (169–177). Therapeutic strategies aimed at manipulating the microbiota, such as with probiotics or prebiotic-directed fermentation amendments of dietary fibers, or microbial product replacement, have emerged to re-establish a metabolic and immune equilibrium and potentially the severity of ARDS by modifying macrophage-endothelium crosstalk (173). These data are indicative of the effects of gut microbiota-generated metabolites on systemic immunity metabolism and might be applicable in inflammatory lung disorders, like sepsis-induced ARDS.

#### 2.6.2 Therapeutic strategies targeting immunometabolism

Immune cells, primarily monocytes-macrophages and ECs, immunometabolism reprogramming is at the heart of ARDS pathogenesis in the context of sepsis. Inhibition of metabolic enzymes and signaling pathways that regulate this reprogramming may present an appealing therapeutic strategy. Inhibitors of glycolysis have been found to modulate macrophage polarization by reprogramming the metabolic shift from M1 phenotype towards an M2 phenotype, consequently down-regulating inflammation and tissue damage (175). Pharmacological compounds targeting key immunometabolic pathways, including the activation of Nrf2 and the inhibition of mitochondrial Complex I by N,N-Dimethylformamide (DMF) or the inhibition of complex I by metformin, are broadly anti-inflammatory and can be used to reset immune homeostasis in inflammation associated with sepsis (177). Moreover, blocking the HIF-1α/lactate dehydrogenase (LDHA) axis to reduce endothelial glycolytic overactivation has been reported to rescue sepsis-induced lung injury and inflammation, indicating the potential of modulating the metabolic pathway. Nanomedicine technologies, such as polymeric nanoparticles carrying metabolically active agents or photoactivatable nano- proteolysis-targeting chimeras (PROTACs) targeting immune-suppressive enzymes, provide alternative tools to fine-tune immune cell metabolism and improve host resistance (178). Moreover, exosomes from mesenchymal stem cells regulate macrophage glycolysis and inflammatory cytokine production by serving as a cell-free therapy for sepsis-induced ARDS (179, 180). However, it remains challenging to temporally and spatially target these metabolic pathways during the sepsis process due to the complexity of immunometabolism networks and the heterogeneity in immune responses. In the future, therapeutic approaches should be directed towards combining immune inhibitors with immunomodulators to achieve a homeostasis that could lead to decreased off-target effects and better outcomes in sepsis-related ARDS. Combining immunometabolic modulation with conventional anti-inflammatory therapies holds significant promise for enhancing treatment efficacy in sepsis-associated ARDS by addressing both metabolic dysregulation and inflammatory cascades. Immunometabolic approaches can reset immune cell function, for example, by inducing stable regulatory T cells (Tregs) through metabolic pathways involving a liver kinase B1 (LKB1)-dependent signaling, which synergize with anti-inflammatory and tissue repair functions (181). Since agents such as metformin and DMF are not only metabolism modulators but also anti-inflammatory agents, a dual effect could be envisioned that would lead to synergy with current available treatments (177). Furthermore, targeting metabolic checkpoints may also reverse immune exhaustion and dysfunction in sepsis, thereby restoring the functional capacity of immune cells and increasing the pathogen clearance rate. However, limitations of this combinatorial logic are the possibility of further immunosuppression (182). The overwhelming heterogeneity among sepsis patients and their dynamic immunometabolic status necessitate a personalized approach for individual patients, utilizing underlying biomarkers such as monocyte human leukocyte antigen DR (HLA-DR) expression and metabolic profiles (182). Furthermore, due to the complexity of metabolic pathways and their interplay with inflammatory signaling, there are inherent risks associated with targeting metabolism; therefore, care must be taken to select targets that do not have counterproductive effects. Nanostructured delivery systems may be part of a targeted co-delivery strategy of metabolic and anti-inflammatory agents, which would contribute to specificity and reduce systemic toxicity (178). Conclusively, integration of immunometabolic regulation with anti-inflammatory therapy appears as an attractive scheme but calls for intensive clinical exploration toward maximum effectiveness, safety, and patient stratification in order to achieve associated ADES.

#### 2.6.3 Emerging approaches for gut-lung axis intervention

**Figure 5:**
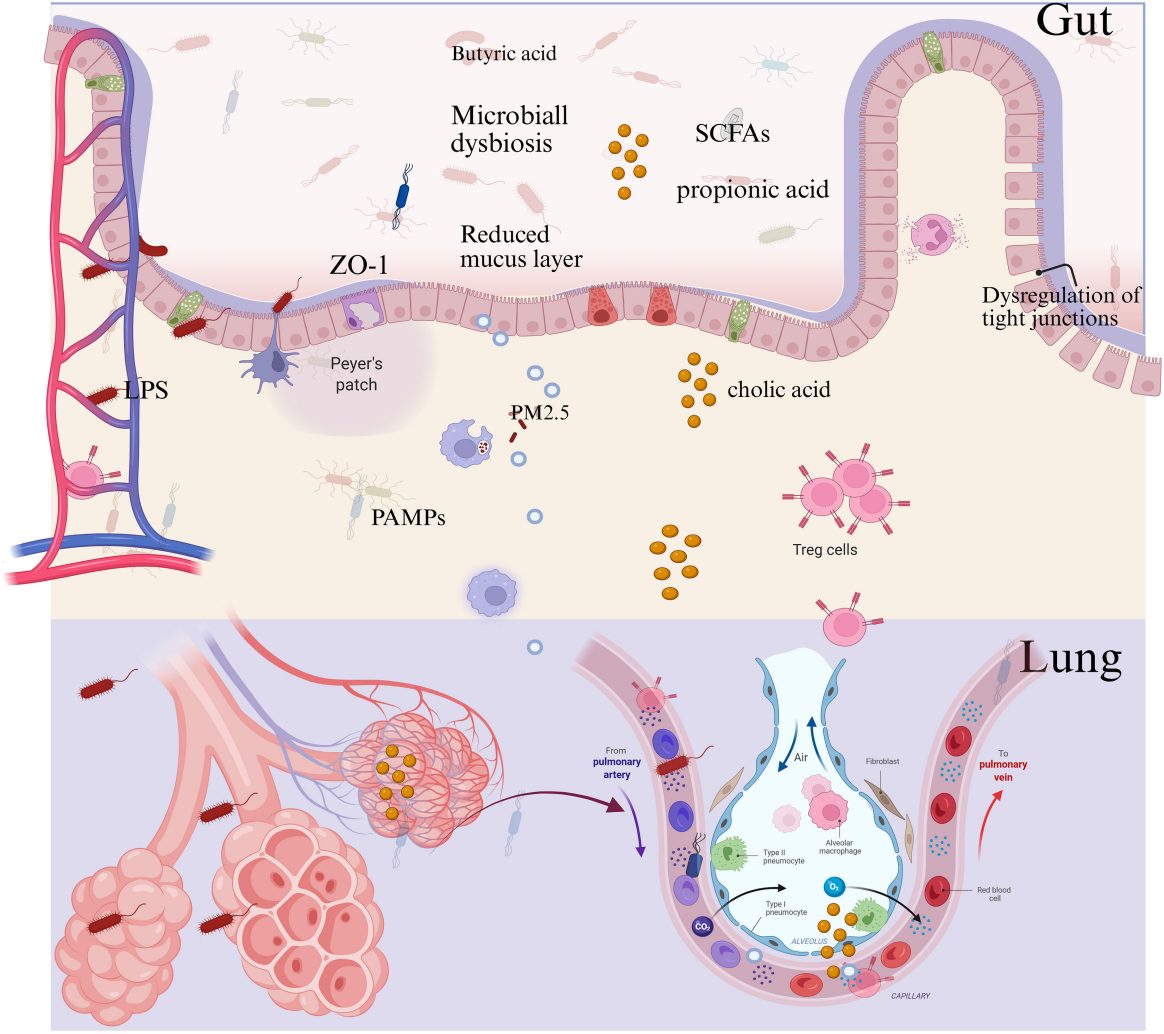
Gut-lung axis intervention [Created with BioRender.com (11)].

Novel treatments addressing the gut-lung axis in sepsis-related ARDS focus on manipulation of the gut microbiota by means of probiotics, restoration of intestinal barrier integrity, and enrichment with beneficial microbial metabolites. Probiotics are promising candidates for maintaining the equilibrium of reconstituted microbial diversity, as seen in the critical respiratory state. In post-acute COVID-19 subjects, supplementing with probiotics increased microbial richness and partially restored immune responses along the gut-lung axis but did not affect gut permeability (183). This indicates that while probiotics can favorably modulate microbial composition and systemic immunity, complementary approaches may be required to reestablish barrier function entirely. The barrier in the gut is critical to preventing harmful bacteria and endotoxins from translocating to the systemic circulation, furthering pulmonary inflammation and injury. Therapeutic interventions that increase mucojunctional tight junctions and maintain mucosal integrity are promising. Traditional Chinese medicine formulae, such as decoction-promoted lung-and-gut barrier integrity in an idiopathic pulmonary fibrosis model through zonula occludens-1(ZO-1) and Claudin-1 upregulation, have been shown to alleviate lung injury by regulating gut-lung crosstalk (184). Supplementation with key microbial metabolites, particularly SCFAs such as butyrate and propionate, represents another promising avenue. SCFAs are generated in the gut through bacterial fermentation of dietary fibers and mediate powerful anti-inflammatory and immunomodulatory activities in the lung (**Figure 5**). They also bind and engage G protein-coupled receptor subtypes, as well as inactivate histone deacetylases, which in turn inhibit proinflammatory cytokines and promote the development of regulatory T cells (185). Dietary approaches that increase fiber consumption have been associated with enrichment in SCFA-producing bacteria and decreased lung inflammation in models of chronic obstructive pulmonary disease and ARDS. Additionally, the direct delivery of SCFAs or their derivatives could circumvent microbial dysbiosis and may represent a more targeted intervention to modulate the lung immune response through the gut-lung axis (186).

Other approaches include FMT, which has been tested to restore eubiosis and immune balance in severe lung infections and inflammatory lung diseases. FMT has been shown to be effective in altering gut microbial composition and reducing systemic inflammation, as well as lung symptoms, in preclinical models of pulmonary fibrosis and bacterial pneumonia (187, 188). However, FMT may cause bacteremia and serious side effects, especially in immunocompromised or critically ill patients. After microbiota transplantation, the risk of transferring pathogens and infection increases significantly, potentially leading to bacteremia, sepsis, and even death. Additionally, lax donor screening, bacterial strains with resistance genes, and viral pathogens can pose safety risks for FMT. Therefore, understanding the specific mechanisms behind FMT-induced bacteremia and clarifying the types and mechanisms of severe side effects is a crucial issue that needs urgent attention (189). Until its mechanisms in immune restoration are well understood and safety confirmed, FMT in sepsis should be considered experimental. Clinical use requires careful consideration of donor selection, safety, and long-term effects.

The sum of all these strategies illustrates that a multifaceted approach to therapeutic modulation is likely necessary to make an impact on the gut-lung axis. Supplementation of probiotics and/or their metabolites, together with a combined factors, may have synergetic effects to rectify the microbiota imbalance restoration and immune homeostasis. Conversely, gut barrier repair therapy prevents the systemic spread of inflammatory mediators. This multifaceted strategy, connecting microbial products as agonists and host factors, induces sepsis-associated ARDS. Dual strategies for co-therapies that simultaneously target drug and metabolism regulation therapy of the drug in combination may be an appealing pattern for the design of therapeutic intervention against sepsis-induced ARDS. Collectively, given the complexity of immunometabolic reprogramming differences between macrophages and ECs in the lung microenvironment, combined modulation of microbial composition with host metabolic pathways may represent more efficient therapeutics. 20(S)-Protopanaxadiol (PPD), derived from ginseng, exhibited its ability to inhibit pulmonary fibrosis by targeting glycolysis and, simultaneously, modulating gut microbiota composition and fecal metabolites, such as those related to the AMPK/STING pathway (187). This dual signature further validates the therapeutic potential of treatment by combined metabolic and microbiota-targeted therapies. Furthermore, the SCFA-mediated alveolar macrophage metabolic switch from glycolysis to OXPHOS was reported to reduce lung immune tone and inflammation, thus suggesting that microbial-derived metabolites exert an influence on lung immune cell function. It is thus conceivable that supplementation with dietary fiber or SCFA analogs could be manufactured to serve as a treatment supplement, which would enhance metabolic homeostasis and immune regulation.

Similarly, treatment with probiotics or prebiotics specific to microbial taxa associated with the production of beneficial metabolites could be personalized based on an individual patient’s microbiome, thereby enhancing precision medicine approaches for therapy in the gut-lung axis. Such personalized approaches may ameliorate the immune-metabolic balance, reduce systemic inflammation, and stimulate lung regenerative mechanisms. Recent data provide evidence that using microbiota-targeted therapies in combination with current drugs or emerging immune modulators might optimize this gut-lung axis interaction. The adjunctive application of probiotics during viral respiratory infections may promote antiviral cytokine responses and attenuate lung inflammation, confirming the broader utility of integrative regimens. In conclusion, rational integration of therapeutic approaches targeting both microbial dysbiosis and host metabolic perturbation emerges as a potential new frontier in the treatment of sepsis-associated ARDS. Precise mechanisms need to be elucidated, and the optimal combination of interventions should be determined, while further clinical trials are warranted to establish efficacy and safety. This multitargeted strategy may ultimately lead to improved clinical outcomes by restoring immune-metabolic equilibrium throughout the gut-lung axis.

## 3 Conclusion

Sepsis-related ARDS poses a significant challenge in critical care medicine, and its complex pathogenesis has long puzzled clinical and basic research fields. Immunometabolic reprogramming plays a pivotal role in sepsis-related ARDS, particularly in driving the inflammatory cascade reaction between macrophages and ECs. The activation state and functional regulation of macrophages directly affect the inflammatory cascade reaction. At the same time, the integrity of the endothelial barrier determines the severity of lung tissue edema and impairment of gas exchange. The dynamic balance between the two constitutes a key node in the pathophysiology of sepsis-related ARDS.

Immunometabolic regulation, a significant research direction in the treatment of sepsis-related ARDS over the past few years, has demonstrated broad prospects for clinical application. The pathogenesis of sepsis-related ARDS is complex, involving a close correlation between immune cell dysfunction and metabolic reprogramming. Recent multi-omics and bioinformatics studies have revealed significant alterations in metabolic pathways in key immune effector cells such as macrophages and ECs during the pathological process, providing a theoretical basis for treatments based on metabolic regulation. Therefore, metabolic regulation combined with anti-inflammatory treatment strategies has emerged as a potential approach to enhance treatment efficacy.

During sepsis and ARDS, immune cells such as macrophages and neutrophils undergo metabolic reprogramming, characterized by increased glycolysis and impaired FAO, leading to uncontrolled inflammatory responses and immune dysfunction. Adjusting the metabolic state of immune cells, such as inhibiting excessive glycolysis or restoring mitochondrial function, can effectively regulate the inflammatory response, thereby reducing tissue damage. Simultaneously, anti-inflammatory drugs can directly inhibit the release of inflammatory mediators. The combination of these two approaches can synergize and enhance the precision and efficacy of treatment.

The emerging understanding of immunometabolic reprogramming in the gut-lung axis presents a strong theoretical model for sepsis-induced ARDS. Immunometabolic reprogramming, as a bridge connecting immune cell function and metabolic status, has emerged as a cutting-edge approach for understanding and intervening in sepsis-related ARDS. The metabolic pathway transition undergone by immune cells under inflammatory stimulation not only regulates the expression and secretion of inflammatory factors but also affects cell survival and repair capabilities. Multiple studies summarized in this article indicate that the switching of energy metabolism modes during metabolic reprogramming in macrophages, such as the transition from OXPHOS to glycolysis, significantly influences the formation of their proinflammatory or anti-inflammatory phenotypes. Simultaneously, it reveals the pivotal role of epigenetics in regulating immunometabolism and the interaction between ECs and macrophages in ARDS. Changes in the metabolic status of ECs play a decisive role in maintaining their barrier function, and metabolic imbalances often lead to cellular dysfunction and exacerbated pulmonary capillary leakage.

The metabolic state of ECs also influences their function and interaction with immune cells. ECs rely on glycolysis as their primary energy source, and abnormalities in their metabolism can lead to dysfunction, affecting vascular permeability and inflammatory responses. Furthermore, complex regulatory networks are formed between cells through the transmission of metabolic products and signaling molecules, facilitating signal transmission between ECs and macrophages and promoting inflammatory cascades. Therefore, regulating metabolic pathways becomes crucial in modulating macrophage-ECs interactions.

It is noteworthy that there are specific differences in the details of immunometabolic regulatory mechanisms across different studies, which may reflect the heterogeneity of sepsis-related ARDS and the complex background of various models and clinical samples. Some studies emphasize the key role of specific metabolic enzymes or signaling pathways, while others focus on the regulatory function of metabolic products as signaling molecules. These different perspectives complement each other, suggesting that future research should focus on multidimensional and multilevel integrated analysis, avoid one-sided interpretations, and strive to construct a more comprehensive and systematic immunometabolic regulatory network.

This review highlights the emerging evidence for immunometabolic reprogramming along the gut-lung axis in sepsis-associated ARDS; however, our understanding primarily comes from preclinical models. To effectively apply these findings to human conditions, clinical studies are necessary. Additionally, while the causal relationships along the gut-lung axis seem plausible, definitively establishing them in patients is challenging. Longitudinal studies that monitor gut integrity, microbial metabolites, and immune cell function in sepsis patients would be highly beneficial. Finally, the promising therapeutic strategies discussed carry the risk of off-target effects that could impact important physiological processes.

Therapeutic strategies based on immunometabolic reprogramming exhibit broad application prospects. By precisely regulating the metabolic states of macrophages and ECs, adjusting their functional polarization, and balancing inflammatory responses, it is expected to alleviate lung tissue damage and improve gas exchange function effectively. Currently, multiple potential drug targets have been discovered, including key metabolic enzymes, transcription factors, and metabolic signaling pathways, providing a theoretical basis and practical foundation for the development of novel therapeutic drugs. However, translating these theoretical achievements into clinically effective and safe treatment regimens still requires overcoming the challenges posed by the complexity of metabolic regulation and individual differences.

Other approaches include FMT, which has been tested to restore eubiosis and immune balance in severe lung infections and inflammatory lung diseases. However, FMT may cause bacteremia and serious side effects, especially in immunocompromised or critically ill patients. After microbiota transplantation, the risk of transferring pathogens and infection increases significantly, potentially leading to bacteremia, sepsis, and even death. Additionally, lax donor screening, bacterial strains with resistance genes, and viral pathogens can pose safety risks for FMT. Until its mechanisms in immune restoration are well understood and safety confirmed, FMT in sepsis should be considered experimental. Clinical use requires careful consideration of donor selection, safety, and long-term effects.

Future research should focus on enhancing the systematic analysis of immunometabolic mechanisms by integrating single-cell multi-omics, metabolomics, and clinical big data to reveal the dynamic relationship between cellular metabolic states and functional phenotypes in depth. Simultaneously, the development of precise metabolic regulation drugs and related biomarkers will facilitate individualized diagnosis and treatment, enhancing the specificity and efficacy of therapy. Interdisciplinary collaboration and the conduct of multicenter clinical trials will also provide solid clinical evidence to support immunometabolic treatment strategies for sepsis-related ARDS. Immunometabolic reprogramming offers a new perspective for understanding the pathological mechanisms of sepsis-related ARDS and paves the way for developing innovative treatment regimens.
